# Efficient screening for enhanced Xe/Kr separation *via* fixed-ligand, variable-metal strategy in metal–organic frameworks

**DOI:** 10.1039/d6ra02043g

**Published:** 2026-07-02

**Authors:** He Zhou, Chunhui Wu, Huimin Xu, Xiaochong Xue, Jinglin Li, Youshi Zeng, Xinxin Chu, Xiaoling Wu, Wei Liu

**Affiliations:** a Shanghai Institute of Applied Physics, Chinese Academy of Sciences Shanghai 201800 China; b University of Chinese Academy of Sciences Beijing 100049 China; c Wuwei Institute of Advanced Energy Gansu Province 733099 China

## Abstract

The separation of xenon (Xe) and krypton (Kr), being an energy-intensive process, has attracted considerable research interest in developing alternative separation methods and materials over the past two decades. Considerable progress has been achieved in discovering sorbent materials with high Xe/Kr selectivity. Due to their tunable pore geometries and interaction strengths, metal–organic frameworks (MOFs) have gained consensus as promising alternatives. For this goal, computational screening methods have been widely adopted to accelerate the discovery of high-performing candidates. However, the computational cost of brute-force screening involving adsorption simulations for millions of structures is prohibitive. Therefore, a practical approach is to partition large-scale screening into manageable subsets focusing on materials with shared chemical features. To conserve computational resources, applying reasonable filters on pore-related geometric features demonstrating significant variation is advisable. Based on these considations, we present a ligand-focused screening strategy employing preliminary geometric filters, including a pore-limiting diameter (PLD) ranging from 3.3 to 8.2 Å and a largest cavity diameter (LCD)/PLD ratio between 1 and 2, together with the restriction to single-ligand-assembled MOFs to facilitate structure–property interpretation. The procedure is exemplified using the ligand 1,1,2,2-tetra(4-carboxyphenyl)ethylene (H_4_TCPE), and experimentally validated the *in silico* workflow through laboratory synthesis and adsorption measurements. The optimal cerium-based framework exhibits a 143% improvement in the adsorbent performance indicator (API), which comprehensively considers selectivity, uptake, and enthalpy, compared to the prior Ca-based analog. Rather than targeting high-throughput discovery, this study demonstrates an efficient and chemically interpretable screening approach for a structurally coherent MOF subfamily. The resulting materials are further contextualized against representative benchmark adsorbents, highlighting their competitive performance and application-relevant advantages.

## Abstract 2

A ligand-oriented screening workflow enables efficient and practical discovery of xenon-selective MOFs, balancing computational cost, synthetic feasibility, and reliable experimental validation.

## Graphical abstract

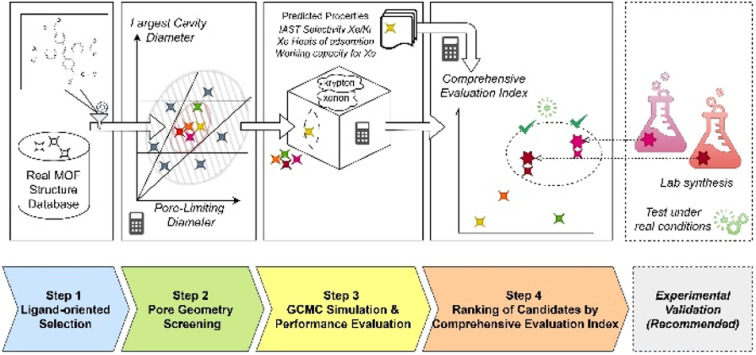

## 1. Introduction

Xenon and krypton are valuable noble gases widely used in medical imaging,^1^ arc lamp plasma,^2^ excimer lasers,^3^ and aerospace industries.^4^ Cryogenic distillation of air separation byproducts (typically containing 20% Xe and 80% Kr by volume), though the most mature technology for noble gas separation, is energy-intensive and expensive due to the low boiling points of Xe and Kr plus their inert chemical nature.^5–9^ These challenges have stimulated increasing attention to adsorption-based alternatives using activated carbon^10^ or other crystalline porous materials, such as zeolite,^7,11^ metal–organic frameworks (MOFs),^12^ covalent–organic frameworks (COFs),^13^ and hydrogen-bonded organic frameworks (HOFs).^14^ Owing to structural tunability as well as the precisely adjustable pore environments, MOFs have emerged as a powerful material class for selective separation of Xe/Kr.^6,8,15,16^

Computational screening techniques have been widely used to identify promising MOFs for Xe/Kr separation.^17–19^ Early studies mainly relied on pore-related geometric descriptors, such as pore size, pore-limiting diameter (PLD), largest cavity diameter (LCD), and pore shape.^20,21^ These parameters have helped researchers build an primary insight on the structure–property relationship between pore geometry and adsorptive performance. As the quantity of models growing from hundreds of experiment-synthesized structures^22–24^ to tens of thousands of hypothetical frameworks,^25–27^ pore-related geometric descriptors alone were no longer sufficient to distinguish materials with similar pore sizes but different host–guest interactions. This motivated the introduction of energy- and adsorption-specific descriptors, including Voronoi energy,^27^ energy histogram,^28^ isosteric heat of adsorption, Henry constants, and GCMC-derived uptake indicators. In the context of evaluating industrial separation processes, combined metrics, such as the separation potential (Δ*Q*),^29^ were also brought up for sorbents applied in a fixed bed adsorption unit. Take another example, the multiparameter adsorbent performance indicator (API) took the xenon isosteric heat of adsorption additionally as consideration in its equation.^24,30^ The improvement of descriptors assisted in revealing more aspects of structural features and provided more efficient criteria for computational screening.

Linker modifications^24^ or metal substitutions^22,31^ were prevailing practices to expand candidate sets. However, virtual structures generated in this way may undergo coordination change or conformational rearrangements, which makes them infeasible to be synthesized and applicable. Besides, it will be complicated for a large-scale screening effort on highly divergent structures to interpret structure–property relationships. Instead, a ligand-oriented screening strategy would be expandable and chemically intuitive to reveal the relationships while save computational resources and ensure the synthetic feasibility.

Following this principle, in this work, we establish a ligand-oriented screening workflow to identify materials for enhanced Xe/Kr separation. This work takes a common tetra-topic 1,1,2,2-tetra(4-carboxyphenyl) ethylene (H_4_TCPE) ligand as an example, filters the MOF family using pore-related geometric descriptors, then applies GCMC simulation to predict Xe/Kr selectivity and obtains a few of top-performing candidates which are subsequently synthesized and tested in laboratory. The experimental validation proved the validity of this workflow and identified a cerium-based framework that outperforms other analogs. This methodology screening from ligand-based subsets can accelerate the exploration of gas sorbent materials, and facilitate the understanding on structure–performance relationship.

## 2. Methods

**Scheme 1:**
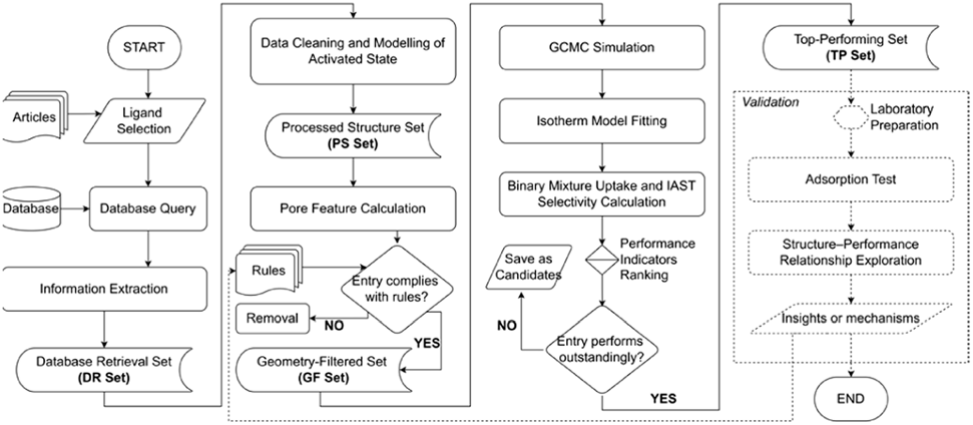
The main workflow of screening xenon-selective ligand-based frameworks from published database.

In this section, the screening workflow is outlined and illustrated as a flowchart in Scheme 1.

### 2.1 Workflow outline

To identify xenon-selective materials within a family of MOFs sharing the same organic linker, a suitable ligand in terms of geometry and functional groups should be selected initially, with those appearing in published outstandingly performed frameworks as a sagacious starting point.

After determining the ligand, constructing database queries to retrieve well-curated information becomes straightforward, especially in meticulously maintained databases such as the Cambridge Structural Database (CSD),^32^ and the Computation-Ready Experimental Metal–Organic Frameworks Database (CoRE MOF DB).^33^ The database retrieval set (DR set) has been acquired, which usually consists redundant and disordered structural information.

Subsequent to the retrieval of crystallographic information files (CIFs), a thorough examination is necessary to obtain solvent- and guest-free, unique structures. These are termed as the processed structure set (PS set), akin to the concept of “computation-ready” in the work of Zhao *et al.*^33^

It is generally accepted that the pore feature parameters like pore-limiting diameter (PLD), largest cavity diameter (LCD), and their ratios reflecting the morphology of the pores in frameworks are highly relative to the selectivity of Xe/Kr.^20,25^ Consequently, the pore features, calculated using the Zeo++ software,^34–37^ form the basis for distinguishing between promising and ordinary materials. The geometry-filtered set (GF set) is further analyzed by Grand Canonical Monte Carlo (GCMC) simulation at a series of pressures under the same temperature to generate isotherms for single component noble gases of xenon, krypton and argon, respectively. Following the fitting of isotherms, binary mixture uptakes as well as the IAST selectivity of Xe/Kr (20 : 80) and Xe/Ar (1 : 99) are designated as performance indicators for evaluation. The top-performing set (TP set) arises from the investigation up to this point.

A laboratory validation is highly recommended. By preparing and testing the best candidates, the structure–performance relationship might provide interesting insights or mechanisms to enrich or adjust the current empirical rules.

### 2.2 GCMC simulation protocols

GCMC simulations were performed by RASPA 2.0 package^38^ on the GF set, which were considered as rigid frameworks, using pure argon, krypton, and xenon as adsorbents.

The 12–6 Lennard-Jones and coulomb potentials (as shown in eqn (1)), which are commonly used by other research groups,^39^ were employed to describe the interactions among inert gases and framework atoms, primarily the van der Waals forces.1
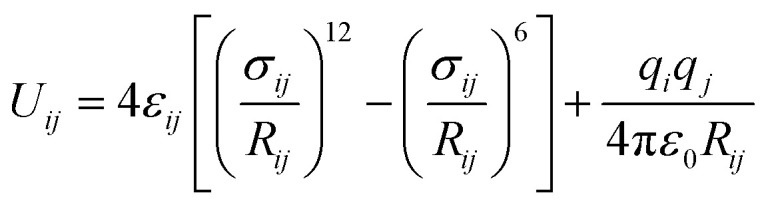


A cutoff radius of 13 Å was applied, along with tail corrections. Each CIF file in the PS set was used to construct a minimal supercell capable of containing a sphere with the cutoff range as its radius, ensuring the minimum-image convention was satisfied.

Lorentz–Berthelot mixing rules were used to calculate cross-term Lennard-Jones parameters. Long-range electrostatic interactions were modeled with a precision of 10^−6^. Framework atoms were modeled using the Universal Force Field (UFF).^40^ Gas molecules (argon, krypton, xenon) were described using the built-in TraPPE force field parameters in RASPA 2.0, respectively.

The multilayer connectivity-based atom contribution (*m*-CBAC) method^41^ for charge assignment on the framework atoms were employed to strike a balance between precision and computational efficiency.

The simulations were run for 10^5^ Monte-Carlo cycles at each pressure, with 10^4^ cycles dedicated to equilibration. A Monte-Carlo cycle consisted of translation, rotation, swapping, and reinsertion steps, all of which were considered in the GCMC calculations. The Peng–Robinson equation of state was used to convert pressure to fugacity. The pressures were set at a series of 10^0^, 10^1^, 10^2^, 5 × 10^2^, 10^3^, 5 × 10^3^, 10^4^, 2.5 × 10^4^, 5 × 10^4^, 7.5 × 10^4^ and 10^5^ Pa, with the temperature fixed at 298 K.

Consequently, the isotherms of three adsorbates were simulated and fitted into adsorption models of the Single-Site Langmuir (SSL),^42^ Single-Site Langmuir–Freundlich (SSLF), Dual-Site Langmuir (DSL),^42^ and Dual-Site Langmuir–Freundlich (DSLF)^43,44^ models. Eqn (2)–(5) describe the four models above, respectively.2
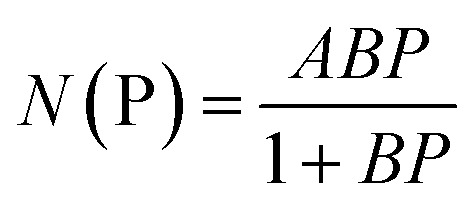
3
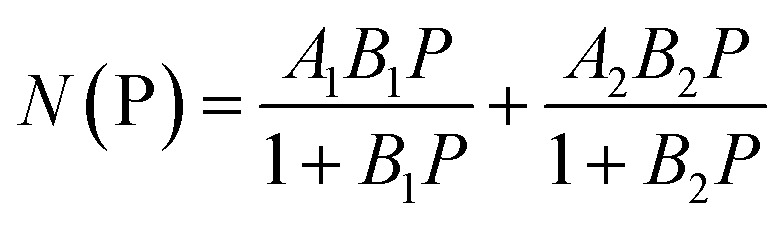
4
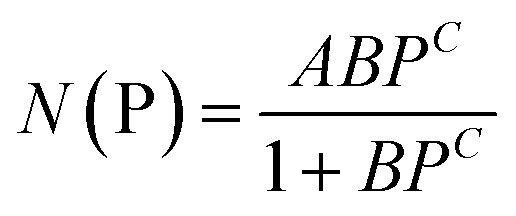
5
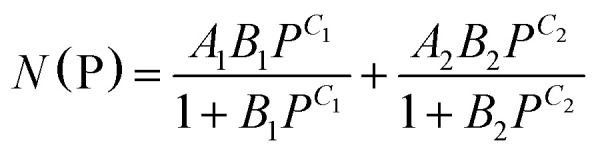


The pressure of gas phase, *P*, is in pascal unit and *N*(*P*) is the amount adsorbed in cm^3^ g^−1^. Subscripts 1 and 2 correspond to two different sites. Parameter *A* (having the same dimension of *N*(*P*)) denotes the saturated capacity. The affinity coefficient, *B*, is in the inverse unit of the *P*^*C*^ term, where *C* is the dimensionless Freundlich parameter related to the surface heterogeneity.

Since the parameters of these models were similar, they were combined in the form of eqn (5) for simplicity. When *A*_2_ = 0, the dual-site models of eqn (3) and (5) recovered to single-site models of eqn (2) and (4). When *C*_1_ = *C*_2_ = 1, the Langmuir–Freundlich models of eqn (4) and (5) recovered to the Langmuir models of eqn (2) and (3).

The best-fitting model, determined by the highest *R*^2^ and reasonable model parameters, was selected for further analysis. It is worth noting that selecting the model with the highest *R*^2^ for each system does not necessarily reflect the true physical behavior, as overfitting may occur. Ideally, GCMC simulations at multiple temperatures would better determine the isosteric heat of adsorption *Q*_st_, thus improving the physical interpretability of the models. Given the inherent uncertainties in assuming all structures are activated and rigid, we pragmatically use the best-fitting model for rapid qualitative screening, sufficient to rank the top candidates for further experimental and theoretical study.

### 2.3 IAST selectivity prediction

Ideal Adsorbed Solution Theory (IAST)^45^ has been widely used to predict the selectivity of gas mixtures based on the pure-component isotherms.^46–49^ IAST predictions for the separation of xenon and krypton were made for a binary mixture with a molar ratio of 20 : 80 in bulk phase. To evaluate the diluted xenon capture from argon as carrier gas, another scenario of IAST predictions adopted a 1 : 99 molar ratio, Xe/Ar binary mixture in bulk phase. Here, the IAST selectivity values in two scenarios were defined as eqn (6) and (7), respectively.6
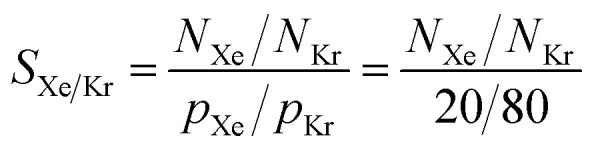
7
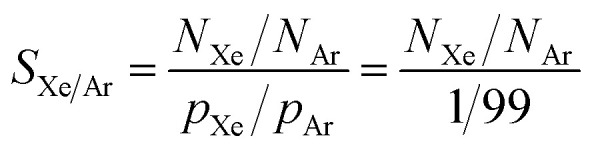


### 2.4 Topological analysis

The geometric and topological analysis of frameworks can performed by the open source ToposPro^50^ program package. Input a CIF file into the program, the network of structure can be simplified by representing metals (or metal clusters) and ligand centroids as nodes, retaining only connections relevant to the primary topology while excluding peripheral atoms.

## 3. Results and discussion

To illustrate how the general screening workflow described in the Methods section operates in practice, a representative ligand family is adopted as a test case in the following subsections. Rather than reiterating methodological details, the focus here is placed on the evolution of structural datasets through each stage of the workflow and on the scientific considerations underlying the corresponding decisions. Intermediate results, selection criteria, and the reasoning for retaining or discarding specific structures are presented to clarify how the workflow functions when applied to real MOF systems. For clarity and readability, only the key outputs and major trends are shown, whereas full data tables, structure lists, and auxiliary figures are provided in the SI.

### 3.1 Rationale for choosing the representative ligand

The ligand 1,1,2,2-tetra(4-carboxyphenyl) ethylene (H_4_TCPE) was selected as the representative example for demonstrating the screening workflow because it provides a structurally versatile and experimentally feasible platform for constructing microporous MOFs. Its molecular geometry as well as its functional groups of carboxylates enable a broad spectrum of coordination behaviors and pore networks, which makes it suitable for illustrating how the datasets evolve throughout the different stages of the workflow.

First, a high degree of coordination diversity is accessible through the four peripheral carboxylate groups of H_4_TCPE. These groups can adopt monodentate, bidentate, bridging, or mixed coordination modes with various metal centers.^51^ Such flexibility has been evidenced by the more than thirty distinct MOF structures retrieved in this study, each exhibiting different three-dimensional network topologies. This diversity ensures that the ligand can effectively represent the range of structural scenarios that the workflow is designed to handle.

Second, the geometric attributes of H_4_TCPE naturally lead to pore apertures relevant to noble-gas adsorption. The typical distance between an ethylene carbon atom and a coordinated metal center is approximately 8 Å, and the angle between adjacent phenyl-carboxylate branches is close to 120°. This arrangement defines an inscribed pore of roughly 6.9 Å in radius, a size suitable for the diffusion and confinement of small inert gases such as Xe and Kr. Consequently, MOFs constructed from this ligand frequently exhibit large pore volumes and accessible channels consistent with efficient gas storage and separation.

Third, chemical and structural stability is commonly observed in H_4_TCPE-based frameworks. The robust metal–carboxylate coordination typically affords good thermal and solvent stability, allowing the materials to preserve porosity under activation or adsorption conditions. The presence of a delocalized π-conjugated backbone further enhances resilience, and several reported frameworks withstand γ-irradiation doses up to 800 kGy,^52^ underscoring their potential suitability for applications involving radioactive noble gases.

Finally, the ligand's intrinsic optical functionality provides additional relevance. As a derivative of the aggregation-induced emission (AIE) chromophore tetraphenylethylene,^53–55^ H_4_TCPE is capable of imparting fluorescence-responsive behavior when incorporated into MOFs.^56,57^ Although optical properties are not the focus of the present study, the multifunctionality of the ligand highlights its broader interest and the value of using it as an illustrative example in evaluating workflow applicability.

Taken together, these structural, geometric, and physicochemical features make H_4_TCPE a suitable model ligand for exemplifying the general screening strategy and for demonstrating how intermediate datasets are transformed into a final set of high-performing candidates.

### 3.2 Database retrieval and construction of the DR set

**Scheme 2:**
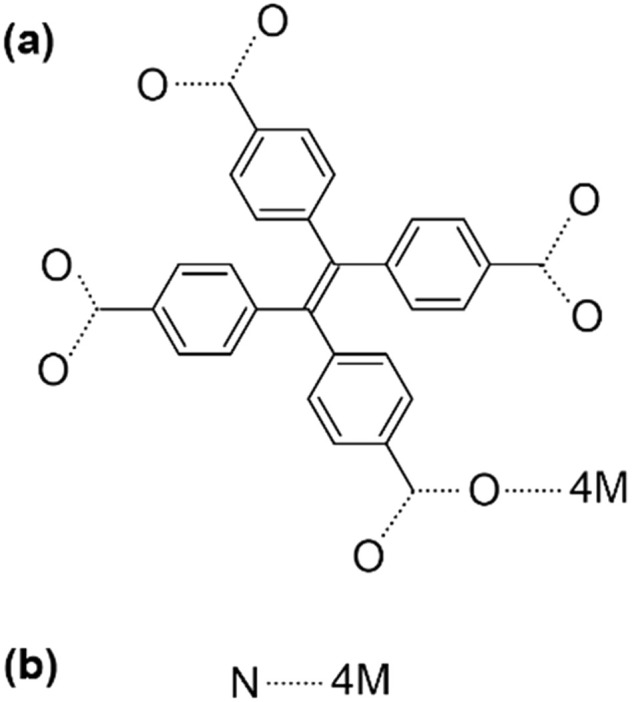
Combination search based on the logical combination of queries: (query a) and (not query b). (a) Query a: represents the H_4_TCPE moiety bonding with any metal atom (symbolized as “4 M”). (b) Query b: represents any nitrogen bonding with any metal. Dotted lines include any type of chemical bonds.

The structure of TCPE^4−^ with one oxygen atom bonding with any metal was sketched as Scheme 2. To keep our study scope concise, structures containing M–N bonds were excluded to prevent the appearance of unwanted ligands—*e.g.*, 2,2′-bipyridine^58^ and its derivatives—from further consideration. This search strategy was implemented in the “Combine Queries” functionality of the ConQuest software.^59^ The query retrieved more than 30 entries from the CSD database, termed as DR set and listed briefly in Table S1.

Structures without three-dimensional information were removed. Those structures that are highly similar to other entries were also ignored because some researchers acquired similar structures from comparable experimental settings. For example, ACUXUU and ACUYAB are almost same structures, with solvent molecules only appears in the former one.^60^ Those six-letter uppercase strings are RefCodes or entry identifiers in the CSD. From each of the following pairs, the former was retained and the latter was removed: (ACUXUU, ACUYAB), (CEDWER, CEDWIV), (ELEDEH, ELEDEH01), (FUXXED, FUXXUT), (KASRAZ, KASRED), (MEVMEI01, MEVMEI). Finally, the DR set was simplified into 31 entries.

### 3.3 Structure cleaning and formation of the PS set

By removing disordered moieties or free solvent in each framework, CIFs exported from the database in the simplified DR set were processed. The gist is that before any calculation or analysis regarding pore, activation of the structures should be considered properly, as the structures in CIFs provided by CSD often contain solvent, adsorbed molecules, as well as distorted atoms. After the removal and adjustment, the activated conformations were imitated as synthesized materials in experimental setups before most testing procedures.

Solvent molecules (mainly water, ethanol and DMF) bonding to the frameworks are usually removed prior to the adsorption tests by heating under vacuum in laboratory settings. It may expose unsaturated open metal sites (OMSs) that can facilitate additional host–guest interactions which enhances the Xe/Kr separation.^8^

The efficacy of such imitation relied highly on the assumption that all conformations hold stable and rigid after removal of unwanted parts, which brought a significant source of error if a solvent-removed framework lost its structural stability supported by the deleted guests and collapsed. Empirically, non-coordinated solvents can be directly removed, while removing coordinated solvents (*e.g.*, M–OH_2_, M–DMF) may create open metal sites (OMS) if the cleaned structure keeps stable. This process was insighted from the data curation work of all-solvent-removed CoRE MOF Database^61^ from Chung *et al.*

### 3.4 Rationale for the geometric filtering criteria

Haldoupis *et al.* introduced the concepts of PLD and LCD to evaluate MOF structures for kinetic separation.^20^ The PLD is defined as the maximum diameter of a sphere that can traverse the entire structure without overlapping with any framework atoms, providing a measure of pore accessibility and diffusional constraints. The LCD represents the largest spherical particle that can be inserted at any point within the material's pores without overlapping with framework atoms, reflecting the local cavity size.

In porous structures where small, agile molecules diffuse alongside larger, slower ones, a higher LCD provides additional “passing lanes”, facilitating the rapid diffusion of smaller molecules and enhancing kinetic separation efficiency. These metrics collectively enable the rational design of materials for gas separation applications by offering insights into pore geometry and molecular transport behavior.

Intuitively, PLD much smaller than krypton is unsuitable for gas diffusion and thus incapable for the application of separation. Similarly, LCD smaller than our concerning gases is inappropriate for adsorbing them.^24^ Thus, we chose 3.3 Å as a lower boundary of PLD and LCD, rather than strictly the kinetic diameter of Kr (3.6 Å). Recent studies highlight that rigid PLD-based predictions misclassified adsorption of C5 compounds as non-adsorptive by neglecting framework flexibility.^62^ Although this effect is less critical for Xe and Kr, which has smaller and simpler geometry, retaining a 0.3 Å margin offsets the bias to a practical extent from for intrinsic flexibility and computational errors.^63^

According to the work of Sikora *et al.*,^25,64^ the highest selectivity of MOFs (including hypothetic MOFs) for Xe/Kr separation were concentrated in the region of structures with LCD slightly greater than 4.1 Å, the diameter of xenon atom. When LCD are much larger than the size of xenon atom, for example LCD > 8.2 Å, which probably represent a low selective material. Besides, the LCD/PLD ratio, reflecting the geometry of pore morphology, exhibits a rule of thumb in predicting the selectivity as well.^25^ Those structures featured with “tube-like pore morphologies” (*i.e.* the LCD/PLD ratio between one and two), are best candidates to selectively capture xenon in gaseous mixture of Xe/Kr. The selectivity decreases drastically as the LCD/PLD ratio exceeds two. Ryan *et al.*^64^ also proposed that an attractive candidate for xenon-selective MOF should have cavities sufficiently large to accommodate Xe atoms but small enough to fit only a single xenon atom.

In this work, the geometric screening criteria were set as (i) 3.3 Å < PLD ≤ LCD < 8.2 Å and (ii) 1 ≤ LCD/PLD ratio ≤ 2, to refine the PS set down to a smaller GF set before time-consuming GCMC simulation.^24^

### 3.5 Screening result of the GF set

PLDs and LCDs of the PS set were calculated *via* a pore analyzer function based on the PoreBlazer^65^ algorithm in the Mercury software.^59^ The values as well as the judgments on the violation of the screening criteria were listed in Table S2.

**Fig. 1:**
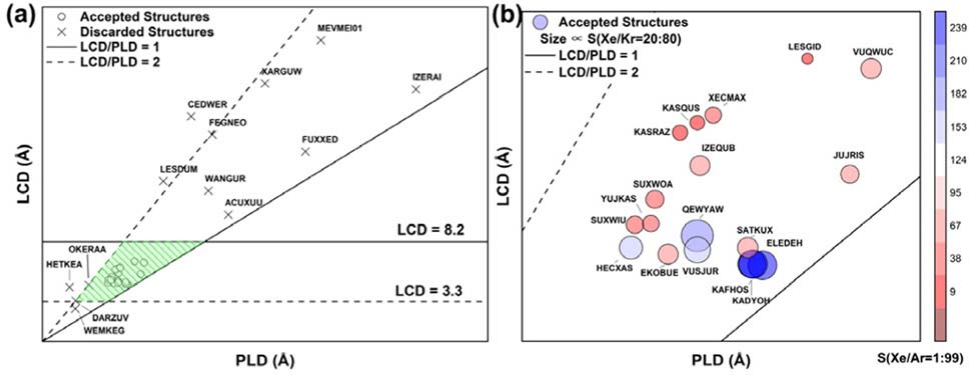
Graphical illustration of the GF set in a coordinate system of PLD as the abscissa and LCD as the ordinate. (a) The region enclosed by the green dashed boundaries is defined by two horizontal lines—a solid line corresponding to LCD = 8.2 and a dashed line corresponding to LCD = 3.3—and two diagonal lines—a solid line defined by LCD/PLD = 1 and a dashed line defined by LCD/PLD = 2. Each marker corresponds to a framework among the PS set. Every open circle (○) inside this region denotes a retained framework; every cross (✗) outside denotes an excluded one. (b) Magnified view of the retention region from (a). The color and size of each open circle correspond to the magnitudes of properties *S*_Xe/Ar_ and *S*_Xe/Kr_, respectively. As indicated by the color bar to the right, hues approaching blue correspond to larger values of *S*_Xe/Ar_, whereas those closer to red indicate smaller *S*_Xe/Ar_. Circle diameter scales directly with the value of *S*_Xe/Kr_.

To delineate the domain of GF set, a coordinate system is illustrated in Fig. 1, with PLD and LCD as the abscissa and ordinate, respectively.

Four straight lines corresponding to the boundaries of screening criteria enclosed a quadrilateral shape filled with green hatching marking the “retention region”. Activated structures in this region (marked by hollow circles) were retained and defined as member of the GF set, whereas those fall outside (marked by crosses) were discarded and excluded from further computation and discussion. Fig. 1b magnified the region of interest highlighted in Fig. 1a, revealing the distribution of the retained structures. The sizes and colors of markers indicated the magnitudes of properties *S*_Xe/Kr_ and *S*_Xe/Ar_, respectively, calculated in following section. Larger and bluer bubbles were gathering at the range of LCD between 4.5–5.0 and the ratio of LCD/PLD close to one, consistent with the empirical rule of “tube-like pore” raised by Sikora *et al.*^25^

Upon topological analysis among the GF set, abundant types of networks were observed and illustrated in Fig. S19 alongside their three-dimensional structures in a parallel arrangement. Although each carboxylate group can theoretically coordinate up to four metals, the geometry features of ligand H_4_TCPE causing steric hindrance and this restricts the connectivity number to moderate values around 1–2. Thus, connectivity numbers (CN) of ligand nodes are predominantly 8 (11 cases) and 6 (5 cases) as listed in Table S8. Interestingly, VUSJUR is special in that its ligand moieties adopt different coordination modes. XECMAX is another unusual case that *N*,*N*-dimethylacetamide (DMA) and water molecules bonding with Zn atoms severely blocks the coordination between metal and ligand carboxylic groups.

This topological features of H_4_TCPE ligand-based MOF family favor the application of kinetic separation of noble gases in terms of the morphology of channels but not cages. It is common that most exhibit one-dimensional channels roughly spanning 1–2 atomic diameters of xenon, permitting xenon atoms to traverse the channels while undergoing frequent collisions with the pore walls. Steric hindrance from the aromatic core and carboxylate groups further restricts three-dimensional cross-linking, favoring straight and regular channels rather than fully three-dimensional cages.

### 3.6 GCMC simulation and IAST selectivity predictions for GF set

These 18 structures of GF set were further analyzed *via* GCMC simulation. The isotherms of argon, krypton as well as xenon at 298 K, 1 atm were collected and fitted into adsorption models to acquire best fitting parameters for the IAST selectivity calculation as detailed in the Section 2.2.

The isotherms were plotted as Fig. S1–S18, with their fitted parameters recorded in Tables S3–S5 and used for calculation of the IAST selectivity values of Xe/Kr and Xe/Ar.

**Fig. 2:**
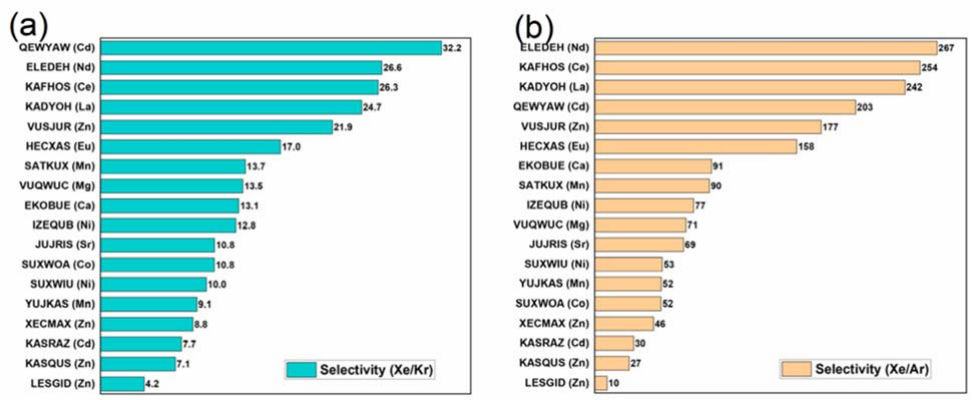
Bar charts of selectivity values for the GF set, (a) for the Xe/Kr (20 : 80) scenario and (b) for the Xe/Ar (1 : 99) scenario.

The selectivity values were tabulated as Tables S6 and S7, and constructed into bar charts in Fig. 2, ranked in descending order.

The bar charts showed that, QEWYAW (Cd),^66^ KAFHOS (Ce),^67^ ELEDEH (Nd)^56^ and KADYOH (La)^57^ and VUSJUR (Zn)^55^ remained the five most xenon-selective candidates in both scenarios, despite minor ranking fluctuations.

EKOBUE (Ca)^52^ and JUJRIS (Sr),^68^ which have been evaluated the application in xenon adsorption in previous studies, fell within the mid-range in both scenarios. Based on another alkaline-earth metal, VUQWUC (Mg)^69^ likewise performed unremarkably. Besides, the divalent transition metals (such as Ni, Co and Mn) afford a rich variety of frameworks; nevertheless, their performance approximated the alkaline-earth-metal analogs. These M^2+^ cations may establish a moderate electrostatic field that enhances adsorption of Xe rather than that of less polarizable Kr and Ar.

It is worth noting that lanthanum (the ground-state electron configuration using noble-gas notation is [Xe]5d^1^6s^2^), cerium ([Xe]4f^1^5d^1^6s^2^) and neodymium ([Xe]4f^4^6s^2^) are lanthanide elements with similar valence electron configurations, which lead to same MOF structures, comparable selectivity values as well as their high thermal and chemical stability.^70^

As group 12 elements with a common oxidation state of +2, Cd and Zn possess d^10^ electronic configuration that yields zero Crystal-Field Stabilization Energy (CFSE),^71,72^ allowing the metal nodes to adopt tetrahedral, octahedral or polynuclear geometries without an energy penalty. Consequently, these cations can generate a rich library of secondary building units (SBUs) and framework topologies.^73–75^

As shown in Fig. 2, KASQUS,^54^ LESDUM,^76^ LESGID,^77^ VUSJUR^55^ and XECMAX^78^ are all frameworks based on metal Zn; the KASRAZ^54^ and QEWYAW^66^ are both Cd-based analogs. The minute differences in pore-characteristic parameters were amplified into substantial xenon-selectivity disparities, because even a narrow PLD margin sufficed to remarkably enhance the interaction between xenon and channel walls.^17^

### 3.7 Limitations of the modeling assumptions and the necessity of experimental validation

Until now, we retrieved from CSD database, cleaned and activated structures, simulated the unary isotherms, predicted the IAST selectivity based on several assumptions that intended to expedite the workflow to an affordable cost of time and calculation resources.

However, the accuracy of selectivity prediction highly relied on the simplified assumptions made as follows.

First, the reported crystal structures were treated ideally by removing guest molecules; they were assumed to be rigid and stable at the ambient conditions as the GCMC simulation settings.

Second, partial atomic charges were assigned based on a multilayer connectivity-based atom contribution (*m*-CBAC) approach since the electrostatic distribution was presumed to exert a negligible effect on the adsorption of single-atom noble gases without dipole or quadruple moment.

Third, the Ideal Adsorption Solution Theory was applied to predict the binary Xe/Kr or Xe/Ar selectivity based on the unary isotherms collected from GCMC simulations, assuming that the IAST holds valid for systems dominated by weak van der Waals interactions.

Although the three assumptions listed above are commonly adopted in similar simulation researches,^31,79^ the actual behaviour of the systems may substantially deviate from these idealized conditions, especially some may exhibit flexible at high temperatures or even at ambient conditions.^31,80^ As these assumptions are inherently interdependent within the whole screening workflow, their cumulative uncertainties may range from causing minor numerical deviations to producing entirely unrealistic results.^17^

In the following sections, it is necessary to evaluate and validate the methodological reliability using several representative MOFs, aiming to ensure that the derived trends remain qualitatively meaningful despite possible quantitative discrepancies.

### 3.8 Experimental validation using the TP set

The five most xenon-selective candidates, namely QEWYAW (Cd),^66^ VUSJUR (Zn),^55^ KAFHOS (Ce),^67^ ELEDEH (Nd)^56^ and KADYOH (La)^57^ constructed as the TP set, were selected as representatives to validate this workflow (Fig. 5).

**Fig. 3:**
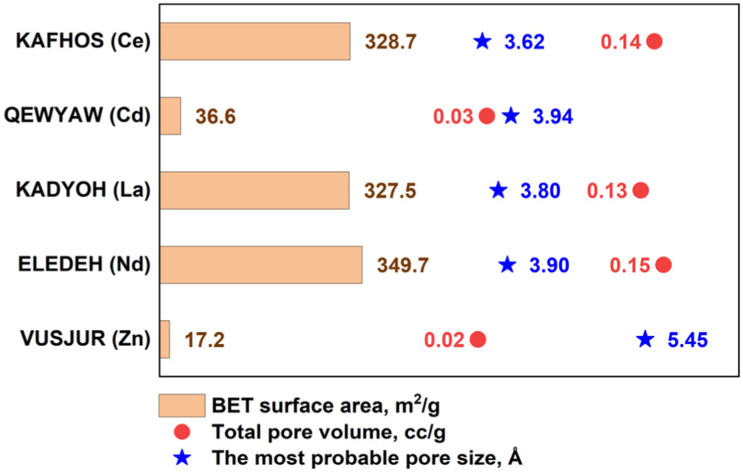
Comparison of BET surface areas (orange bars), pore volumes (red solid circles) and the most probable pore sizes (blue solid stars) among representative materials.

The preparation, identification *via* powder X-ray diffraction, and adsorption tests of these materials are detailed in the SI. The experimental isotherms of xenon at 273 K, 298 K and 313 K, as well as that of krypton and argon at 298 K were plotted in Fig. S20–S24. The Brunauer–Emmett–Teller (BET) surface areas, pore volumes and the most probable pore sizes were compared in Fig. 3.

**Fig. 4:**
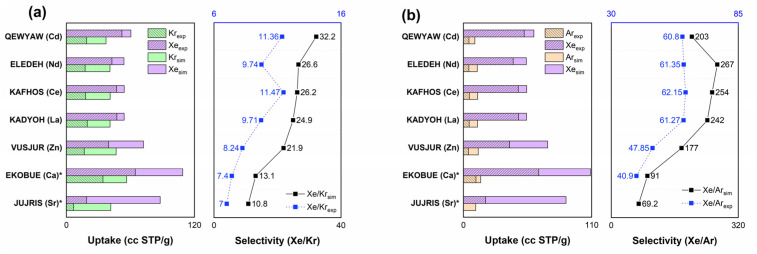
Comparison of pure component uptakes and IAST selectivity between GCMC simulated (uptakes: bars without hatching; selectivity: solid black lines) and experimental tested results (uptakes: bars with hatching; selectivity: dotted blue lines). All values are at 1 bar and 298 K, except values of EKOBUE (Ca) are at 293 K instead. Experiment data of EKOBUE (Ca) and JUJRIS (Sr) are from their original references. (a) For the scenario of Xe/Kr (molar ratio = 20 : 80) separation; (b) for the scenario of Xe/Ar (molar ratio = 1 : 99) separation.

The uptake of adsorbates as well as the selectivity estimated *via* IAST based on measured isotherms (parameters in Table S9), juxtaposed with those from GCMC simulated results, were summarized in Fig. 4 (or tabulated form in Table S10). The experiment results of EKOBUE (Ca)^52^ and JUJRIS (Sr)^68^ were reported from their original references.

**Fig. 5:**
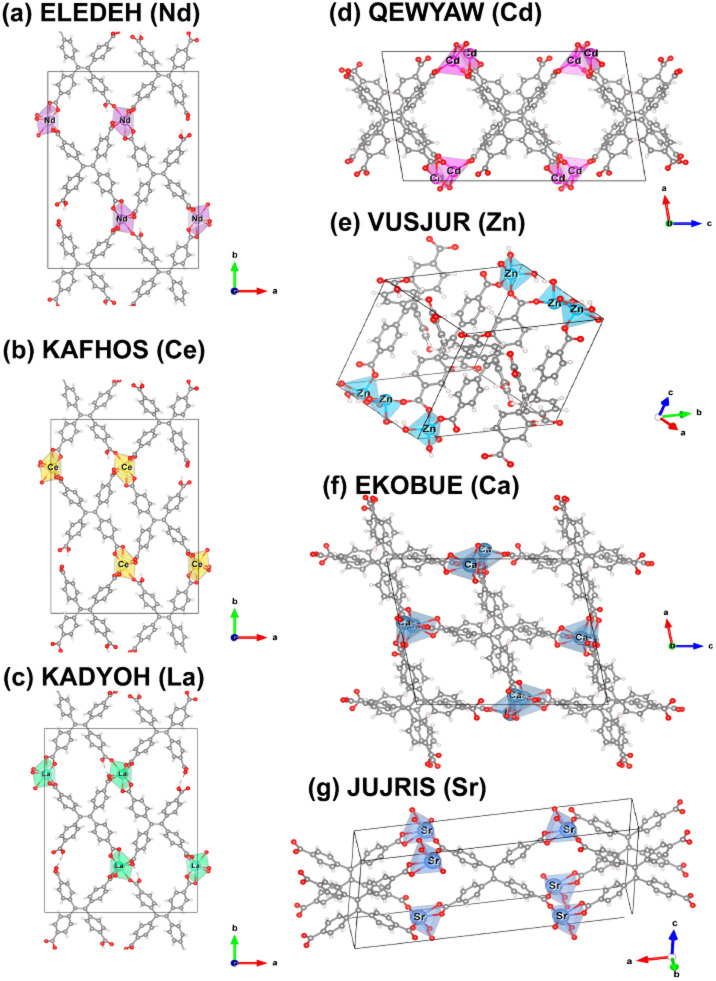
The unit cell crystal structures of (a–e) frameworks from TP set as well as structures of (f) EKOBUE (Ca) and (g) JUJRIS (Sr). Color code: Nd, pink; Ce: yellow; La, mint; Cd: magenta; Zn, cyan; Ca, teal; Sr, blue; O, red; C, gray. H atoms are omitted for clarity.

**Fig. 6:**
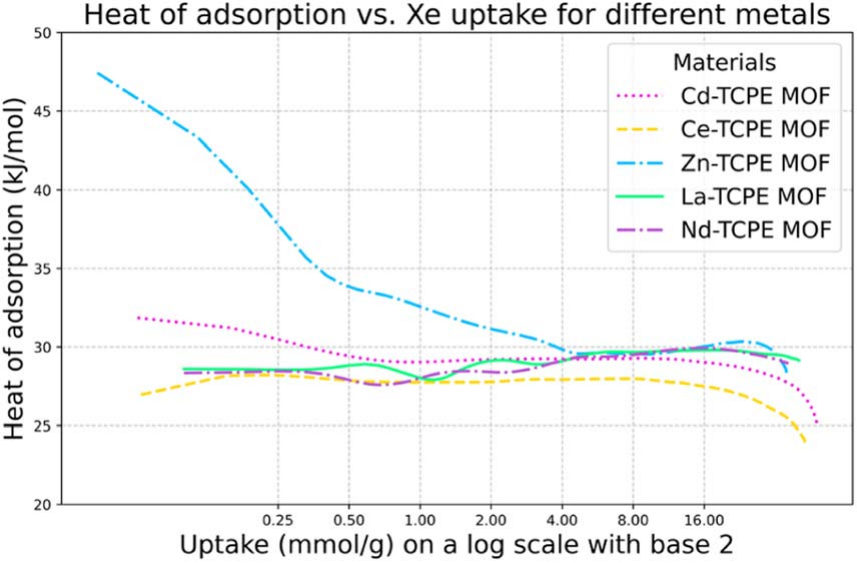
Isosteric heats of adsorption *versus* xenon uptake at 298 K for the five most xenon-selective MOFs on the same coordinate axes of log_2_ scale.

The isosteric heat of adsorption *Q*_st_, which varies with loading, quantifies the average enthalpy change associated with adding one more molecule to the adsorbent at a given loading level. It serves as an indicator of the adsorption affinity, and it reflects the intensity of interaction between the adsorbates and the frameworks. The xenon isosteric heats of adsorption for the representatives were obtained and illustrated against xenon uptake amount (see Fig. 6, and more details in Fig. S25).

### 3.9 Discussion on the discrepancies and exceptions

The reasons of discrepancies between measured results and predicted ones from the screening workflow is discussed below in the respects of uptake, selectivity, adsorptive enthalpy and a comprehensive indicator integrated three of them.

#### 3.9.1 Discrepancies of gas uptake values

From the rankings of performance summarized from Fig. 4, it is obvious to observe a systematic overestimation of gas uptake, especially for the JUJRIS (Sr), EKOBUE (Ca) and VUSJUR (Zn).

The removal of disordered and guest molecules often causes a slight contraction of pores. Without further geometry optimization by the *ab initio* calculation, overestimation of adsorption behaviors that are sensitive to the pore size parameters is inevitable yet acceptable with small error.

As the Xiong *et al.* stated in their work,^68^ the reported heat of adsorption *Q*_st_ exhibiting an increasing function with respect to xenon uptake. This could be ascribed to that as more xenon adsorbed in pore, the flexible cavities become larger and easier for more xenon to be accommodated within. According to their thermogravimetric analysis, the material loses 30% of its mass around 400 °C, indicating complete activation. However, the authors used 200 °C in vacuum for activation before the xenon adsorption test. Such insufficient activation might cause the unreleased solvent molecules blocking the cavities. Thus, the tested uptake amount of less than 20 cm^3^ Xe per g is much less than the predicted 87.9 cm^3^ g^−1^ and the real capacity.

EKOBUE (Ca) was presumed to be non-accessible for gas with kinetic diameter larger than its PLD of 3.60. However, the breakthrough experiment of previous study has successfully measured the breakthrough points at different residence times and detected both xenon and krypton from the effluent,^52^ which proved the efficacy of dynamic separation despite the counterintuitive fact that larger adsorbates should have been filtered out by packed column bed. This indicates that the framework is flexible in pore structures to allow the entrance of xenon and krypton atoms. With a large LCD of 4.79, it can therefore uptake more adsorbates than other candidates.

VUSJUR (Zn) has PLD (3.82) and LCD (4.83) values similar to those of EKOBUE (Ca). However, the BET value of VUSJUR (19.0 m^2^ g^−1^) is much lower than that of EKOBUE (143.4 m^2^ g^−1^). Therefore, the gas uptake values for both predicted and measured fall behind those of EKOBUE.

#### 3.9.2 Discrepancies of selectivity values

Apart from gas uptake, the selectivity values are overestimated systematically as well. Generally speaking, the rankings are in good agreement with that of prediction, with acceptable range of errors.

KAFHOS (Ce) outperforms the other two lanthanide analogs and a cadmium one in both scenario of Xe/Kr and Xe/Ar separation, which was qualitatively underestimated. This can be explained by the limitations of the simplistic force field employed in simulation. In contrast to other metals, cerium exhibits variable oxidation states and a more complex local electronic environment. These features can enhance short-range polarization and induction interactions with highly polarizable adsorbates such as Xe. However, such effects are not explicitly included in the non-polarizable LJ 12–6 plus fixed-charge model adopted in our GCMC simulations. Consequently, the adsorption affinity of Xe on the KAFHOS (Ce) tends to be underestimated relative to experiment, whereas metals with more stable oxidation states exhibit smaller deviations.

As listed in Table S2, the LCDs of QEWYAW (Cd) and VUSJUR (Zn) are 4.96 and 4.83, respectively, leaving space not very ample to accommodate guest molecules. What's worse, the PLDs of both (3.82) are smaller than the kinetic diameter of xenon (3.96) yet larger than that of krypton (3.80) and argon (3.54), making cavities the only place in cells to settle xenon molecules if the framework is rigid. A slight dwelling of cavities due to stimuli from adsorption or working conditions could deteriorate the predicted selectivity values from 32.2 of QEWYAW (Cd) and 21.9 of VUSJUR (Zn) radically to 11.4 and 8.2, respectively.

#### 3.9.3 Comparison on the heats of adsorption curve *versus* xenon uptake

In the case of isosteric heats of adsorption, as Fig. 6 shows, the dotted magenta line representing QEWYAW (Cd) and the dash-dot blue line for VUSJUR (Zn) drop drastically from the low-loading to higher region. This drastic decline can be attributed to the occupation of unsaturated open metal sites around their pores. Water molecules (two bonding with Cd1 and another two bonding with Cd2 in the CIF of QEWYAW; one bonding with Zn2 in the CIF of VUSJUR) can be activated to create such open metal sites and enhance the xenon affinity in low coverage. After the strongest adsorption sites at the channel corners near the OMSs were occupied by the first few xenon molecules, subsequent molecules filled the remaining small pores, primarily along the channel edges with weaker intensity of interaction.

The dash-dot purple line representing ELEDEH (Nd), and solid green line representing KADYOH (La) remain relatively flat especially at high loading region; however, compared with the other four, the dashed yellow line for KAFHOS (Ce) shows the most significant downward trend as uptake increases.

From Fig. 3, the pores of KAFHOS (Ce) are distributed around 3.6 Å, smaller than 3.8 Å of KADYOH (La) and 3.9 Å of ELEDEH (Nd). Given the same mass of materials of three lanthanide analogs, KAFHOS (Ce) has same total volume of pores with others, but the void is contributed mainly from narrower cavities, where KADYOH (La) and ELEDEH (Nd) have wider pores. As the guest coverage become higher, narrow cavities with diameters smaller than the atomic size of xenon would be more thermodynamically unfavorable to adsorb more xenon. Thus, heat of adsorption for KAFHOS (Ce) falls behind others.

#### 3.9.4 Evaluation of candidates for industrial application using adsorbent performance indicator

The materials identified through the screening process are all derived from previously reported literature. Given that the synthesis methods employed in this study are readily scalable, it can be inferred that expanding the synthesis to a larger scale would not present significant challenges. However, transitioning from laboratory research to industrial application necessitates an evaluation of the adsorption and separation performance within processes such as pressure swing adsorption (PSA) and temperature swing adsorption (TSA).

Therefore, a versatile factor, adsorbent performance indicator (API) developed by Wiersum *et al.*^30^ was introduced as eqn (8).8
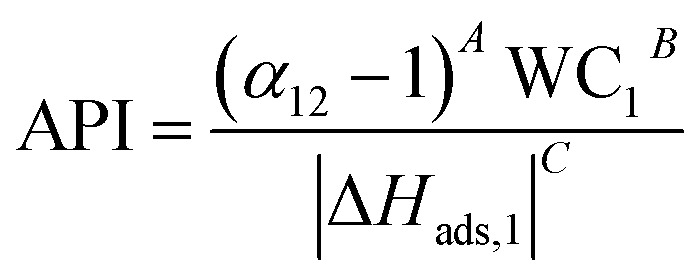


In eqn (8), *α*_12_ represents the selectivity parameter of gas component 1 (Xe) over 2 (Kr), WC denotes working capacity, and Δ*H*_ads,1_ corresponds to the xenon isosteric heat of adsorption indicating the easiness of regeneration in a process.^30^

**Fig. 7:**
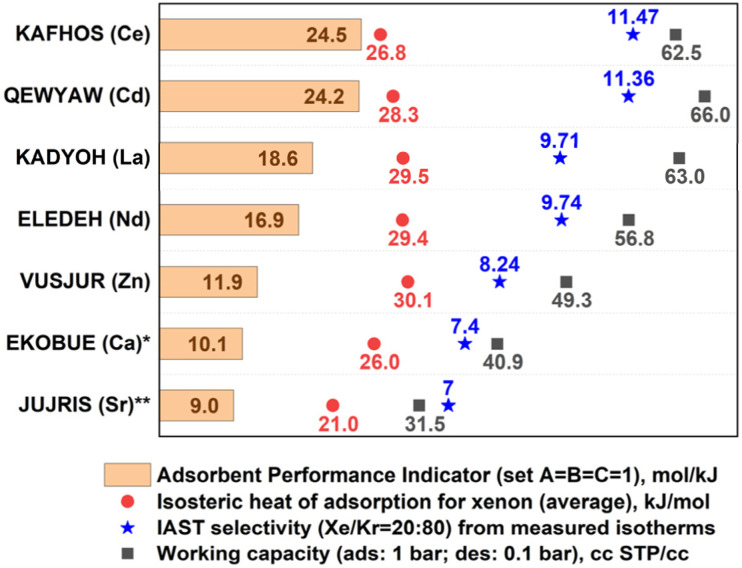
Comparison of APIs (orange bars), isosteric heats of adsorption (red solid circles), IAST selectivity values of Xe/Kr (20 : 80) (blue solid stars) and working capacity (setting adsorption pressure at 1 bar and desorption pressure at 0.1 bar) (gray solid squares) among the representative materials. EKOBUE (Ca) data from original reference at 293 K. JUJRIS (Sr) data from original reference at 298 K.

To evaluate at the same condition, the exponents of *A*, *B* and *C* were set as 1 to keep equal weights. As for working capacity (per unit volume of sample), at this early discovery stage, the adsorption and desorption pressures were set temporarily at 1 bar and 0.1 bar, respectively. The volumetric unit were converted from the gravimetric value by the particle density estimated from the activated model in the PS set if not given in the original references. The isosteric heat of adsorption values were averaged over the range of xenon loadings. Detailed data were tabulated in Table S11, and generated the APIs ranked as Fig. 7.

Exhibiting the highest selectivity, near-maximum working capacity, and lowest Xe isosteric heat of adsorption among reported H_4_TCPE ligand-based MOFs, KAFHOS (Ce) emerges as the optimal candidate, demonstrating promising potential for industrial applications.

#### 3.9.5 Performance benchmarking and practical validation of the optimal candidate KAFHOS

**Fig. 8:**
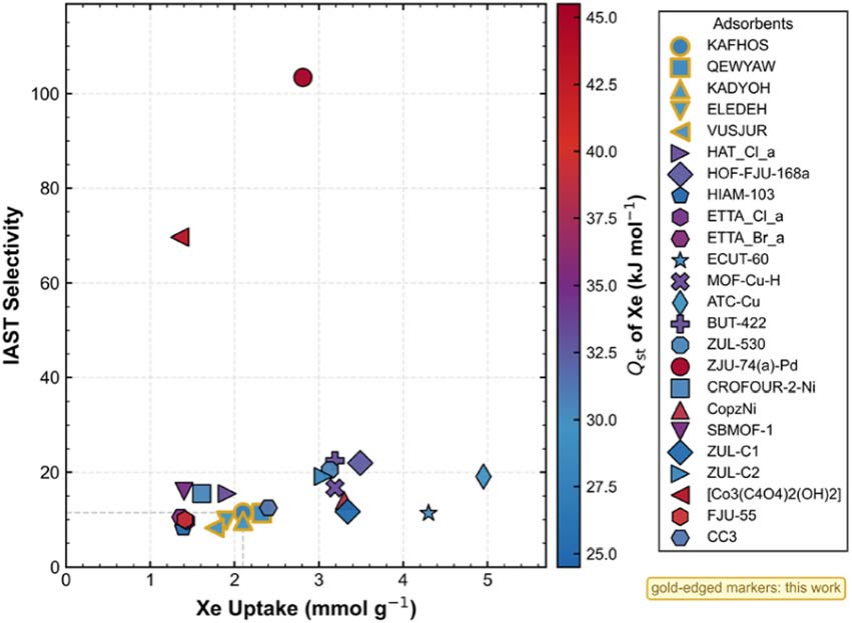
Xe/Kr (20 : 80) IAST selectivity *versus* Xe uptake (at 298 K, 1 bar) for representative adsorbents. Colors denote isosteric heat of adsorption (*Q*_st_, kJ mol^−1^) from blue (low) to red (high). Markers with gold-edged highlighting the five most xenon-selective candidates in this work, and upper-right ones perform superior.

To contextualize the separation performance, representative best-performance materials were summarized along with the five most xenon-selective MOFs in this work. As illustrated in Fig. 8 (Xe uptake *vs.* Xe/Kr IAST selectivity plot), KAFHOS occupies a moderate position within the landscape of high-performance adsorbents, offering a balanced combination of capacity and selectivity comparable to CC3 (ref. 81) and ZUL-C1,^82^ while surpassing several materials such as ETTA_Cl_a, ETTA_Br_a,^83^ HIAM-103 (ref. 84) and FJU-55 (ref. 85) in both separation metrics. However, it remains inferior to recent benchmark MOFs such as ATC-Cu,^86^ ECUT-60,^87^ BUT-422,^88^ and ZUL-530.^89^

Beyond static adsorption performance, practical deployment in nuclear off-gas treatment demands robustness under ionizing radiation. We therefore evaluated the radiolytic stability of KAFHOS under both β and γ irradiation up to 200 kilogray—a cumulative dose well-established in the literature as a standard assessment criterion for materials intended for nuclear waste remediation.^90^ Post-irradiation PXRD analysis (Fig. S36) confirms retention of crystallinity and framework integrity, with no structural degradation observed. This radiation resistance, combined with the inherent chemical stability of the KAFHOS framework, substantiates its operational viability in low-activity nuclear off-gas environments where radiation exposure is significant but manageable.

Finally, to validate the practical separation capability under dynamic flow conditions relevant to engineered processes, we conducted multicomponent breakthrough experiments. Using a ternary gas mixture (0.10% Xe, 0.40% Kr, 99.5% Ar) at 298 K and near-atmospheric pressure, the breakthrough curves (Fig. S37) demonstrate clear temporal separation of the three components, with Xe retained longest followed by Kr, while Ar elutes rapidly as the carrier gas. This dynamic validation confirms that KAFHOS can effectively separate Xe/Kr mixtures under continuous flow, bridging the gap between computational screening and real-world application.

## 4. Conclusions

In this study, we developed a simple yet effective workflow for identifying high-performance MOFs with superior Xe/Kr selectivity within structural families of given ligand. The H_4_TCPE ligand and its derived framework family were selected as a demonstration case for the workflow validation. Among the evaluated frameworks, the Ce-based analog KAFHOS^67^ exhibited the highest adsorbent performance index (API = 24.5) and record IAST selectivity for Xe/Kr (20 : 80 mixture) of 11.47 under ambient conditions, surpassing the previous reported promising candidate of Ca-based EKOBUE in the same ligand family. Although initially predicted to rank third computationally, KAFHOS demonstrated superior experimental performance compared to higher-ranked candidates. These findings not only validate the workflow's efficacy but also underscore the necessity of experimental verification, since rigid-structure simulations may not fully capture actual material performance. Machine learning potentials have demonstrated significant advances in simultaneously enhancing computational efficiency and prediction accuracy.^91^ Therefore, incorporating these potentials into the current workflow represents a promising approach for improving the reliability and accuracy of future adsorption simulations.

This workflow enables rapid screening of optimal Xe/Kr separation materials based on ligand selection. Systematic variation of ligands with comparable sizes, coordination modes, or functional groups enables compilation of structurally comparable subsets to elucidate structure–property relationships. The workflow is readily transferrable to other gas separation systems where pore size and morphology are critical determinants.

The workflow primarily utilizes open-source tools and avoids dependence on costly Density Functional Theory (DFT) calculations or specialized computational chemistry expertise. This accessibility enables adoption by small research groups with constrained budgets or limited computational experience. While our demonstration used the commercial CSD as data source, open-source alternatives like CoRE MOF^61^ and the Topologically Based Crystal Constructor (ToBaCCo)^92^ serve as excellent substitutes. These databases frequently offer pre-processed structures, ensuring both reliability and user-friendliness.

In the current study, the workflow employs geometric features of framework cavities as the primary screening criteria and adsorptive performance as the secondary criterion. Future computational screening studies should incorporate practical application factors such as large-scale synthetic feasibility, stability, recyclability, material lifetime, and overall cost.^26^ This multi-objective evaluation strategy for material selection and optimization would help bridge the gap between computational predictions and deployable sorbent materials.

## Author contributions

He Zhou: methodology, investigation, data curation, software, writing – original draft, writing – review and editing, formal analysis and visualization. Chunhui Wu: investigation and writing – review and editing. Huimin Xu, Xiaochong Xue, and Jinglin Li: investigation and resources. Youshi Zeng, and Xinxin Chu: project administration and funding acquisition. Xiaoling Wu, and Wei Liu: conceptualization, resources, supervision, project administration, funding acquisition and writing – review & editing.

## Conflicts of interest

There are no conflicts to declare.

## Supplementary Material

RA-016-D6RA02043G-s001

## Data Availability

The authors confirm that the data supporting the findings of this study are available within the article and its supplementary information (SI). Supplementary information: this material provides intermediate results of the exemplary ligand H_4_TCPE-based screening process, including (a) information of the database retrieval set, (b) pore geometric parameters of the processed structures set, (c) Grand Canonical Monte Carlo simulated isotherms and IAST selectivity predicted values, topological details for structures of the geometry-filtered set, (d) laboratory validation procedures, adsorption tests and evaluation of the top-performing set, and (e) comparison with high-performance materials. See DOI: https://doi.org/10.1039/d6ra02043g.
