# Supplementary material for: Efficient screening for enhanced Xe/Kr separation via fixed-ligand, variable-metal strategy in metal–organic frameworks
Source: RSC Adv. 2026 Jul 2;16(34):33407–18. doi: 10.1039/d6ra02043g (PMC13326657; doi:10.1039/d6ra02043g)
Supplement: RA-016-D6RA02043G-s001 [file RA-016-D6RA02043G-s001.pdf]

Supplementary Information (SI) for RSC Advances.
This journal is © The Royal Society of Chemistry 2026

Supplementary Information

Efficient Screening for Enhanced Xe/Kr Separation via Fixed-Ligand, Variable-Metal Strategy in Metal–Organic Frameworks

He Zhou,[a,b] Chunhui Wu,[a,c] Huimin Xu,[a] Xiaochong Xue,[a] Jinglin Li,[a] Youshi Zeng,[a] Xinxin Chu,[a] Xiaoling Wu,[a,c,*] and Wei Liu[a,c,*]

[a] Shanghai Institute of Applied Physics, Chinese Academy of Sciences, Shanghai 201800, China
[b] University of Chinese Academy of Sciences, Beijing 100049, China
[c] Wuwei Institute of Advanced Energy, Gansu Province 733099, China
* Corresponding Authors: Xiaoling Wu and Wei Liu. E-mail: wuxiaoling@sinap.ac.cn; liuwei@sinap.ac.cn

Contents

Section S1. Retrieved Data, Process and Analysis for the Exemplary Ligand H_4TCPE-based Screening Process..2
S1.1 Information on Database Retrieved Set....2
S1.2 Pore geometric parameters of the Processed Structures Set....4
S1.3 GCMC simulated isotherms and IAST Selectivity values of the Geometry-Filtered Set....5
S1.4 Topological details for structures of interest dataset. 13
Section S2. Laboratory Validation....14
S2.1 Materials....14
S2.2 Preparation of Five Most Xenon-Selective Materials....14
S2.3 Adsorption Test....14
S2.3.1 Measured Isotherms....15
S2.3.2 Heat of Adsorption for Xenon....17
S2.3.3 Pore Volume and Pore Size Distribution....18
S2.4 Powder X-ray Diffraction Test....20
S2.5 Evaluation of the Adsorbent Performance Indicator....22
S2.6 Comparison of KAFHOS performance with other representative materials....23
S2.7 Gas Breakthrough Experiments....24
References....25

Section S1. Retrieved Data, Process and Analysis for the Exemplary Ligand H_4TCPE-based Screening Process

S1.1 Information on Database Retrieved Set

Table S1. Initial dataset exported from ConQuest Software. These discarded entries are marked in red font and their "Name" column is marked with an asterisk.

No.	Name	a (Å)	b (Å)	c (Å)	α	β	γ	Space group	formula	CCDC Deposition Number	DOI of references
**1**	ACUXUU	26.06	19.40	11.10	90	97.2	90	$C2/c$	$(C_{30}H_{24}Ag_4O_{12})_n$	1494374	10.1021/acscatal.6b03404
**2**	ACUYAB*	11.25	18.51	26.38	90	97.3	90	$P2_1/n$	$(C_{30}H_{16}Ag_4O_8)_n$,2$(C_{11}H_{12}O)$,2.5(CH_2Cl_2)	1494395	10.1021/acscatal.6b03404
**3**	CEDWER	29.98	29.98	29.98	90	90	90	$Pm\bar{3}m$	$(C_{96}H_{88}O_{68}Zr_{12})_n$,0.55$(C_8H_{40}O_{32}Zr_6)$	2159612	10.1039/D2DT01108E
**4**	CEDWIV*	29.93	29.93	29.93	90	90	90	$Pm\bar{3}m$	$(C_{102}H_{100}O_{68}Zr_{12})_n$,0.52$(C_{16}H_{24}O_{32}Zr_6)$	2159615	10.1039/D2DT01108E
**5**	DARZUV	10.25	9.74	30.29	90	93.2	90	$P2_1/n$	$(C_{60}H_{38}O_{24}U_3)_n$,2$(H_2O)$	2072729	10.1021/acs.cgd.2c00016
**6**	EKOBUE	16.28	11.90	18.99	90	100.6	90	$P2_1/n$	$(C_{30}H_{22}Ca_2O_{11})_n$	2031144	10.1021/acs.inorgchem.0c02841
**7**	ELEDEH	20.42	27.28	5.42	90	90	90	$P2_12_12$	$(C_{32}H_{23}NdO_9)_n$	2058221	10.1016/j.inoche.2021.108550
**8**	ELEDEH01*	20.42	27.28	5.42	90	90	90	$P2_12_12$	$(C_{32}H_{23}NdO_9)_n$	2058221	10.1016/j.inoche.2021.108550
**9**	FEGNEO	30.13	30.13	30.13	90	90	90	$Im\bar{3}m$	$(C_{90}H_{108}O_{72}Zr_{12})_n$,18$(C_3H_7NO)$,40$(H_2O)$	2111432	10.1021/acs.inorgchem.2c00545
**10**	FUXXED	27.59	27.59	13.67	90	90	120	$P\bar{3}$	$(C_{33}H_{31}NNi_2O_{13})_n$	1416026	10.1021/jacs.5b07925
**11**	FUXXUT*	27.78	27.78	13.62	90	90	120	$P\bar{3}$	$(C_{34}H_{33}NNi_2O_{13})_n$,0.5$(C_3H_7NO)$,0.33$(C_8H_8O)$, 0.5$(CH_4O)$, 0.5$(H_2O)$	1410111	10.1021/jacs.5b07925
**12**	HECXAS	11.78	26.84	21.71	90	94.8	90	$P2_1/n$	$(C_{67}H_{47}Eu_3N_2O_{20})_n$	2126479	10.1021/acs.inorgchem.2c01332
**13**	HETKEA	10.55	11.10	14.92	72.1	80.3	65.3	$P\bar{1}$	$(C_{30}H_{21}LaO_{10})_n$	2179472	10.1016/j.jssc.2022.123568
**14**	IZEQUB	27.27	11.54	13.97	90	90	90	$Pmna$	$(C_{60}H_{36}Ni_3O_{20})_n$,7n$(H_2O)$	1502000	10.1039/C6RA22971A
**15**	IZERAI	26.25	26.25	11.24	90	90	120	$P6/mmm$	$(C_{90}H_{60}Ni_6O_{32})_n$,n$(H_2O)$	1502001	10.1039/C6RA22971A
**16**	JUJRIS	27.95	6.88	11.09	90	98.5	90	$C2/m$	$(C_{36}H_{34}N_2O_{12}Sr_2)_n$	1974289	10.1016/j.jssc.2020.121337
**17**	KADYOH	20.41	27.22	5.40	90	90	90	$P2_12_12$	$(C_{32}H_{23}LaO_9)_n$	1944290	10.1021/acs.inorgchem.0c02604
**18**	KAFHOS	20.38	27.25	5.42	90	90	90	$P2_12_12$	$(C_{32}H_{23}CeO_9)_n$	2027647	10.1016/j.jssc.2020.121820
**19**	KASQUS	13.70	17.15	21.46	90	90.0	90	$P2_1/c$	$(C_{30}H_{20}O_{10}Zn_2)_n$,4n$(C_5H_{11}NO)$	866864	10.1021/ja209327q
**20**	KASRAZ	11.41	16.59	26.04	90	95.3	90	$P2_1/c$	$(C_{39}H_{39}Cd_2NO_{11})_n$,n$(C_5H_{11}NO)$	866865	10.1021/ja209327q
**21**	KASRED*	11.49	18.52	25.73	90	96.3	90	$P2_1/c$	$(C_{37}H_{35}Cd_2NO_{11})_n$,2n$(C_5H_{11}NO)$	866866	10.1021/ja209327q
**22**	LESDUM	63.65	18.96	41.93	90	111.2	90	$C2/c$	$(C_{150}H_{104}N_4O_{66}Zn_{14})_n$,$H_2O$	2070619	10.1002/adfm.202106925
**23**	LESGID	21.77	13.63	17.65	90	90	90	$P2_1/c$	$(C_{50}H_{64}N_4O_{14}Zn_2)_n$	2143083	10.1002/advs.202200850
**24**	MEVMEI*	43.98	43.98	43.98	90	90	90	$Fm\bar{3}m$	$(C_{90}H_{48}O_{32}U_4{}^{4-})_n$,4$(C_2H_8N^+)$	1575362	10.1021/acs.cgd.7b01525
**25**	MEVMEI01	43.84	43.84	43.84	90	90	90	$Fm\bar{3}m$	$(C_{90}H_{48}O_{32}U_4{}^{4-})_n$,4$(C_2H_8N^+)$	2065409	10.1021/acs.inorgchem.1c00863
**26**	OKERAA	11.13	11.56	14.92	68.6	78.6	61.3	$P\bar{1}$	$(C_{64}H_{42}O_{22}Tb_3{}^{-})_n$,2$(H_2O)$,$H_3O^+$	2178563	10.1039/D0CC06478E
**27**	QEWYAW	14.08	10.99	23.57	90	98.5	90	$P2_1/c$	$(C_{33}H_{29}Cd_2NO_{12})_n$	2103510	10.1039/D2DT03388G
**28**	SATKUX	18.13	18.13	12.59	90	90	90	$I\bar{4}$	$(C_{60}H_{38}Mn_4O_{20})_n$	2210095	10.1039/D1DT03349B
**29**	SUXWIU	11.42	14.85	13.19	90	90	90	$P222_1$	$(C_{30}H_{20}Ni_2O_{10})_n$,2$(C_4H_9NO)$	2026282	10.1002/anie.202012019
**30**	SUXWOA	11.40	14.96	13.26	90	90	90	$P222_1$	$(C_{30}H_{20}Co_2O_{10})_n$,2$(C_4H_9NO)$	2026283	10.1002/anie.202012019
**31**	VUQWUC	26.24	24.69	14.05	90	90	90	$Cmca$	$(C_{38}H_{36}Mg_2N_2O_{11})_n$	2011498	-
**32**	VUSJUR	11.81	11.82	14.79	81.8	69.7	73.4	$P\bar{1}$	$(C_{33}H_{27}NO_{12}Zn_3)_n$,$C_3H_7NO$	1984426	10.1039/D0CE01092H
**33**	WANGUR	30.09	30.09	30.09	90	90	90	$Im\bar{3}m$	$(C_{114}H_{92}O_{64}Zr_{12})_n$,18$(C_3H_7NO)$,12$(H_2O)$	2105643	10.1016/j.cej.2021.134057
**34**	WEMKEG	8.35	12.46	13.80	90	90	90	$Pbam$	$(C_{30}H_{24}Co_2O_{12})_n$,n$(H_2O)$	833060	10.1039/c2ce26761f
**35**	XARGUW	30.40	30.40	73.65	90	90	120	$R32$	$(C_{180}H_{96}O_{64}U_8)_n$	2053178	10.1007/s10562-021-03544-5
**36**	XECMAX	15.47	15.51	18.50	68.9	82.9	75.6	$P\bar{1}$	$(C_{144}H_{130}N_6O_{44}Zn_8)_n$,4$(C_4H_9NO)$,4$(H_2O)$	2127630	10.1039/D2DT00264G
**37**	YUJKAS	11.62	13.10	25.03	90	97.2	90	$P2_1/c$	$(C_{33}H_{25}Mn_2NO_{10})_n$,$C_3H_7NO$,$C_2H_3N$	1951489	10.1021/acsami.9b22410

S1.2 Pore geometric parameters of the Processed Structures Set

Table S2. Pore-limiting diameters and largest cavity diameters as well as the violation of geometry-filtered criteria for each structure in the PS Set.

No.	RefCode	Metal	PLD (Å)	LCD (Å)	LCD/PLD	Violation of geometry-filtered criteria
1	ACUXUU	Ag	9.39	10.42	1.11	LCD>8.2
2	CEDWER	Zr	7.51	18.52	2.47	LCD>8.2, LCD/PLD>2
3	DARZUV	U	1.68	3.37	2.01	PLD<3.3, LCD/PLD>2
4	EKOBUE	Ca	3.60	4.79	1.33	
5	ELEDEH	Nd	4.31	4.69	1.09	
6	FEGNEO	Zr	8.58	17.02	1.98	LCD>8.2
7	FUXXED	Ni	13.28	15.62	1.18	LCD>8.2
8	HECXAS	Eu	3.32	4.85	1.46	
9	HETKEA	La	1.37	4.47	3.26	PLD<3.3, LCD/PLD>2
10	IZEQUB	Ni	3.84	5.6	1.46	
11	IZERAI	Ni	18.86	20.72	1.10	LCD>8.2
12	JUJRIS	Sr	4.97	5.52	1.11	
13	KADYOH	La	4.23	4.7	1.11	
14	KAFHOS	Ce	4.24	4.7	1.11	
15	KASQUS	Zn	3.82	5.99	1.57	
16	KASRAZ	Cd	3.69	5.9	1.60	
17	LESDUM	Zn	6.11	13.18	2.16	LCD>8.2, LCD/PLD>2
18	LESGID	Zn	4.65	6.57	1.41	
19	MEVMEI01	U	14.06	24.76	1.76	LCD>8.2
20	OKERAA	Tb	2.33	4.66	2.00	PLD<3.3
21	QEWYAW	Cd	3.82	4.96	1.30	
22	SATKUX	Mn	4.20	4.85	1.15	
23	SUXWIU	Ni	3.35	5.06	1.51	
24	SUXWOA	Co	3.50	5.29	1.51	
25	VUQWUC	Mg	5.13	6.48	1.26	
26	VUSJUR	Zn	3.82	4.83	1.26	
27	WANGUR	Zn	8.38	12.39	1.48	LCD>8.2
28	WEMKEG	Co	1.66	2.71	1.63	PLD<3.3, LCD<3.3
29	XARGUW	U	11.24	21.19	1.89	LCD>8.2
30	XECMAX	Zn	3.94	6.06	1.54	
31	YUJKAS	Mn	3.47	5.07	1.46	

S1.3 GCMC simulated isotherms and IAST Selectivity values of the Geometry-Filtered Set

Table S3. Fitting parameters for argon isotherms at 298 K. The model types and definitions of parameters can be found in Manuscript Section 2.3. Note: because all the R^2 values approach 1 and the long strings of nines following the decimal point might obscure the assessment of fit quality, as an alternative goodness-of-fitness parameter, *GoF* was defined as $GoF = -\lg\left(1 - R^2\right)$. For example, $GoF(0.99999) = 5$. The higher GoF value indicates R^2 closer to 1.

**RefCode**	**Model type**	GoF	A_1	A_2	B_1	B_2	C_1	C_2
EKOBUE	SSLF	5.1	79.73	0	2.17E-03	1	1.024	1
ELEDEH	SSLF	4.4	52.30	0	2.60E-03	1	1.026	1
HECXAS	SSLF	5.4	59.83	0	2.09E-03	1	0.998	1
IZEQUB	SSLF	5.2	146.88	0	8.53E-04	1	0.998	1
JUJRIS	SSLF	5.3	142.79	0	9.34E-04	1	0.994	1
KADYOH	SSLF	5.1	62.48	0	2.39E-03	1	0.998	1
KAFHOS	SSLF	4.4	73.56	0	2.15E-03	1	0.980	1
KASQUS	SSLF	5.3	222.32	0	4.24E-04	1	1.012	1
KASRAZ	SSL	5.5	245.74	0	3.59E-04	1	1	1
LESGID	SSLF	5.2	92.59	0	7.20E-04	1	1.023	1
QEWYAW	SSLF	5.2	82.44	0	1.38E-03	1	0.993	1
SATKUX	SSLF	5.0	81.07	0	1.22E-03	1	1.013	1
SUXWIU	SSLF	4.9	159.85	0	5.71E-04	1	0.994	1
SUXWOA	SSLF	4.7	174.69	0	5.13E-04	1	0.998	1
VUQWUC	SSLF	5.2	271.39	0	4.73E-04	1	0.991	1
VUSJUR	SSLF	5.1	78.92	0	1.83E-03	1	1.008	1
XECMAX	SSLF	4.9	234.89	0	4.51E-04	1	0.993	1
YUJKAS	SSLF	6.0	114.00	0	1.09E-03	1	1.005	1

Table S4. Fitting parameters for krypton isotherms at 298 K.

**RefCode (Metal)**	**Model type**	GoF	A_1	A_2	B_1	B_2	C_1	C_2
EKOBUE (Ca)	DSLF	5.7	82.49	26.83	1.76E-03	3.76E-02	1.323	0.996
ELEDEH (Nd)	DSLF	5.0	43.42	9.83	1.12E-02	9.96E-02	1.193	1.031
HECXAS (Eu)	SSLF	5.0	59.77	0	1.59E-02	1	0.979	1
IZEQUB (Ni)	DSLF	4.8	93.82	63.60	2.21E-03	6.81E-03	0.935	1.053
JUJRIS (Sr)	DSLF	4.5	93.96	57.02	2.04E-05	1.11E-02	1.845	1.068
KADYOH (La)	DSLF	4.8	45.54	9.56	1.54E-02	7.48E-02	1.103	1.017
KAFHOS (Ce)	DSLF	5.3	43.88	10.16	1.29E-02	9.00E-02	1.155	1.026
KASQUS (Zn)	DSLF	4.7	58.68	51.88	1.05E-05	6.22E-03	2.071	1.065
KASRAZ (Cd)	SSLF	4.4	557.89	0	6.58E-04	1	0.961	1
LESGID (Zn)	SSLF	4.9	318.82	0	5.59E-04	1	1.018	1
QEWYAW (Cd)	DSLF	6.7	44.66	7.87	2.05E-03	4.40E-02	1.485	1.633
SATKUX (Mn)	DSL	6.0	96.65	31.56	2.74E-03	9.86E-03	1	1
SUXWIU (Ni)	DSL	6.6	108.94	44.70	1.52E-03	5.76E-03	1	1
SUXWOA (Co)	DSLF	5.5	45.32	20.56	5.19E-04	1.91E-02	1.530	1.006
VUQWUC (Mg)	DSL	5.0	152.49	90.09	1.04E-03	5.05E-03	1	1
VUSJUR (Zn)	DSLF	8.2	63.60	8.77	7.30E-03	8.79E-02	1.172	0.902
XECMAX (Zn)	SSLF	4.3	119.49	0	4.72E-03	1	0.967	1
YUJKAS (Mn)	DSLF	5.8	75.91	64.68	1.19E-04	9.70E-03	1.546	0.994

Table S5. Fitting parameters for xenon isotherms at 298 K.

RefCode (Metal)	Model type	GoF	A_1	A_2	B_1	B_2	C_1	C_2
EKOBUE (Ca)	DSL	4.7	96.05	66.80	0.171	4.06E-03	1	1
ELEDEH (Nd)	DSLF	5.5	20.51	48.17	5.67E-02	0.911	0.433	1.147
HECXAS (Eu)	DSL	3.5	274.16	47.71	1.02E-04	0.432	1	1
IZEQUB (Ni)	DSLF	4.7	144.71	51.54	2.66E-02	0.105	0.673	1.229
JUJRIS (Sr)	DSL	5.3	209.27	87.36	4.73E-04	0.107	1	1
KADYOH (La)	DSLF	5.2	23.98	47.91	5.44E-02	0.849	0.411	1.178
KAFHOS (Ce)	DSLF	5.5	7.81	52.24	1.32E-03	0.860	1.259	1.084
KASQUS (Zn)	DSLF	5.5	118.19	24.39	1.06E-02	5.96E-02	1.163	0.881
KASRAZ (Cd)	SSLF	4.2	194.50	0	1.39E-02	1	0.995	1
LESGID (Zn)	DSLF	3.9	164.02	12.97	1.01E-04	2.24E-02	1.960	1.694
QEWYAW (Cd)	DSLF	6.3	54.92	6.53	0.385	0.610	1.130	0.779
SATKUX (Mn)	DSLF	4.7	73.48	12.18	0.114	3.91E-02	0.886	2.091
SUXWIU (Ni)	DSLF	4.7	89.79	12.37	4.35E-02	7.08E-02	0.930	1.204
SUXWOA (Co)	DSLF	4.6	86.27	12.10	2.52E-02	0.261	1.126	1.092
VUQWUC (Mg)	DSLF	5.7	107.87	100.91	1.89E-02	6.74E-02	0.657	1.115
VUSJUR (Zn)	DSLF	4.9	25.35	57.18	0.124	0.427	0.560	1.166
XECMAX (Zn)	DSLF	4.9	76.88	14.02	5.30E-02	5.96E-02	0.929	1.366
YUJKAS (Mn)	DSLF	5.4	11.98	80.17	2.35E-02	7.90E-02	1.811	0.940

Table S6. IAST selectivity of Xe/Kr (20:80 molar ratio) at 1 bar, 298 K based on Table S4 and S5. Uptake amount predicted for binary adsorption at 1 bar in cc STP/g. Selectivity (20:80) Xe over Kr predicted values at 1 bar.

RefCode (Metal)	Model of Kr	Model of Xe	Kr uptake	Xe uptake	Selectivity
QEWYAW (Cd)	DSLF	DSLF	6.19	49.86	32.23
ELEDEH (Nd)	DSLF	DSLF	6.61	43.97	26.60
KAFHOS (Ce)	DSLF	DSLF	6.73	44.20	26.25
KADYOH (La)	DSLF	DSLF	7.08	43.70	24.68
VUSJUR (Zn)	DSLF	DSLF	10.04	54.99	21.92
HECXAS (Eu)	SSLF	DSL	8.65	36.81	17.02
SATKUX (Mn)	DSL	DSLF	14.30	49.03	13.71
VUQWUC (Mg)	DSL	DSLF	20.54	69.09	13.46
EKOBUE (Ca)	DSLF	DSL	20.18	66.00	13.08
IZEQUB (Ni)	DSLF	DSLF	17.56	56.12	12.79
JUJRIS (Sr)	DSLF	DSL	19.51	52.61	10.79
SUXWOA (Co)	DSLF	DSLF	14.69	39.55	10.77
SUXWIU (Ni)	DSL	DSLF	15.86	39.70	10.01
YUJKAS (Mn)	DSLF	DSLF	20.27	46.26	9.13
XECMAX (Zn)	SSLF	DSLF	17.51	38.33	8.75
KASRAZ (Cd)	SSLF	SSLF	20.10	38.48	7.66
KASQUS (Zn)	DSLF	DSLF	20.88	37.01	7.09
LESGID (Zn)	SSLF	DSLF	14.78	15.32	4.15

Table S7. IAST selectivity of Xe/Ar (1:99 molar ratio) at 1 bar, 298 K based on Table S3 and S5. Uptake amount predicted for binary adsorption at 1 bar in cc STP/g. Selectivity (1:99) Xe over Ar predicted values at 1 bar.

RefCode (Metal)	Model of Ar	Model of Xe	Ar uptake	Xe uptake	Selectivity
ELEDEH (Nd)	SSLF	DSLF	7.64	20.62	267.07
KAFHOS (Ce)	SSLF	DSLF	8.15	20.89	253.83
KADYOH (La)	SSLF	DSLF	8.16	19.95	241.98
QEWYAW (Cd)	SSLF	DSLF	7.73	15.89	203.49
VUSJUR (Zn)	SSLF	DSLF	9.82	17.52	176.59
HECXAS (Eu)	SSLF	DSL	7.76	12.35	157.56
EKOBUE (Ca)	SSLF	DSL	13.35	12.28	91.04
SATKUX (Mn)	SSLF	DSLF	8.19	7.40	89.50
IZEQUB (Ni)	SSLF	DSLF	10.42	8.12	77.16
VUQWUC (Mg)	SSLF	DSLF	11.12	7.99	71.07
JUJRIS (Sr)	SSLF	DSL	10.95	7.66	69.23
SUXWIU (Ni)	SSLF	DSLF	7.95	4.23	52.67
YUJKAS (Mn)	SSLF	DSLF	10.65	5.58	51.88
SUXWOA (Co)	SSLF	DSLF	8.12	4.24	51.76
XECMAX (Zn)	SSLF	DSLF	9.42	4.36	45.79
KASRAZ (Cd)	SSL	SSLF	8.33	2.55	30.32
KASQUS (Zn)	SSLF	DSLF	9.33	2.53	26.83
LESGID (Zn)	SSLF	DSLF	6.90	0.67	9.60

Figures S1-18 are GCMC simulated isotherms of argon, krypton and xenon at 298K for the 18 candidates.

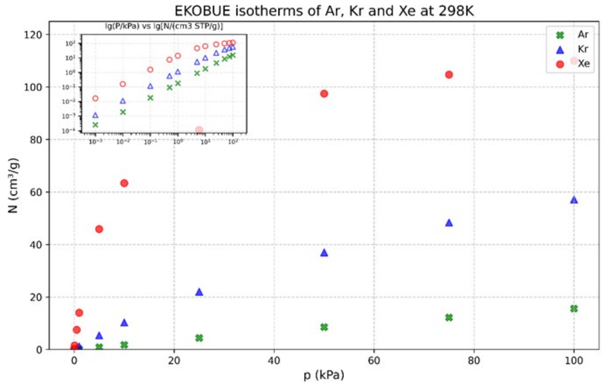

Figure S1. The GCMC simulated isotherms of adsorbates at 298K for EKOBUE (Ca).

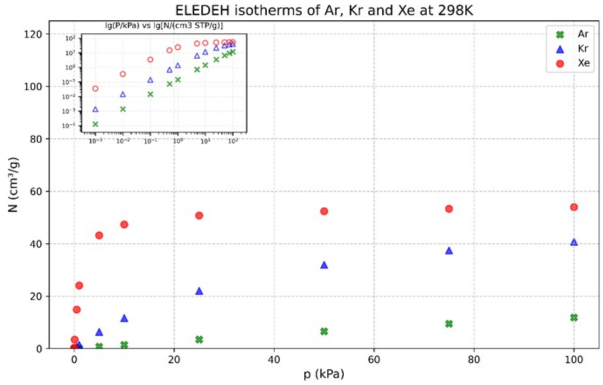

Figure S2. The GCMC simulated isotherms of adsorbates at 298K for ELEDEH (Nd).

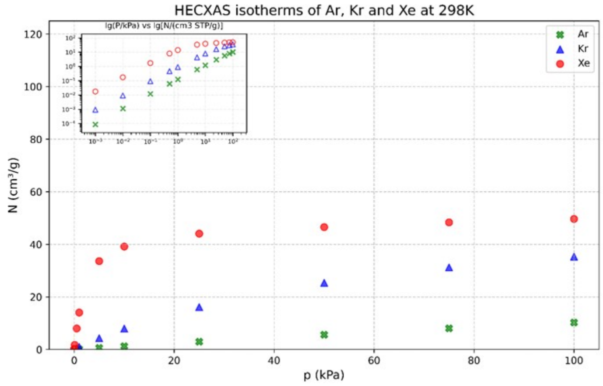

Figure S3. The GCMC simulated isotherms of adsorbates at 298K for HECXAS (Eu).

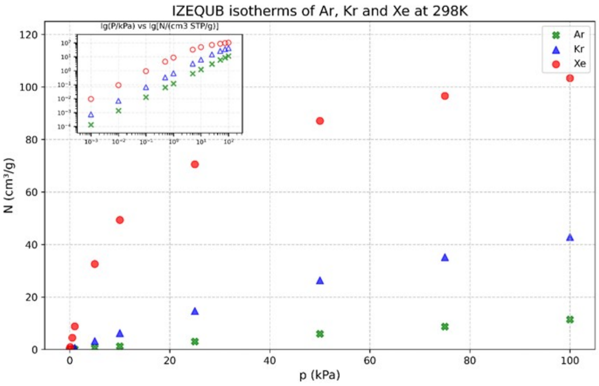

Figure S4. The GCMC simulated isotherms of adsorbates at 298K for IZEQUB (Ni).

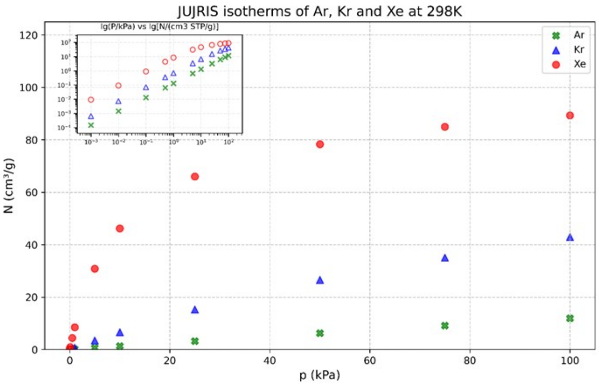

Figure S5. The GCMC simulated isotherms of adsorbates at 298K for JUJRIS (Sr).

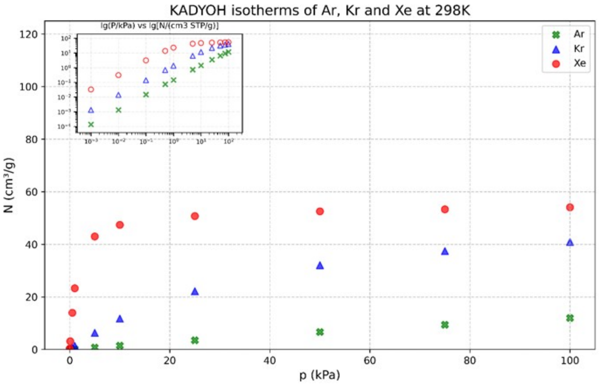

Figure S6. The GCMC simulated isotherms of adsorbates at 298K for KADYOH (La).

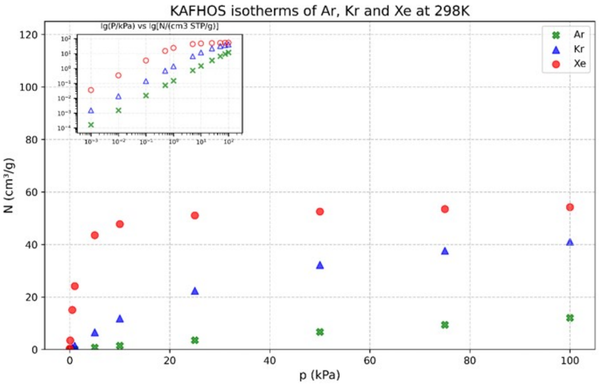

Figure S7. The GCMC simulated isotherms of adsorbates at 298K for KAFHOS (Ce).

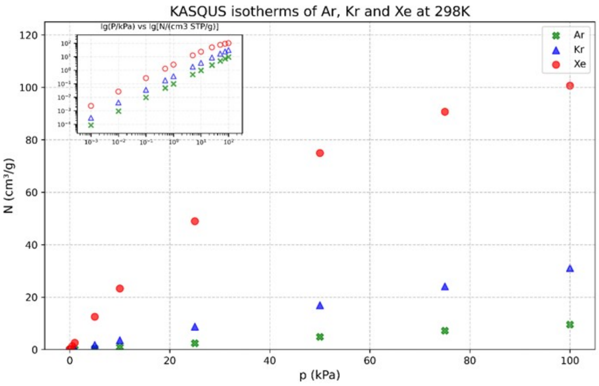

Figure S8. The GCMC simulated isotherms of adsorbates at 298K for KASQUS (Zn).

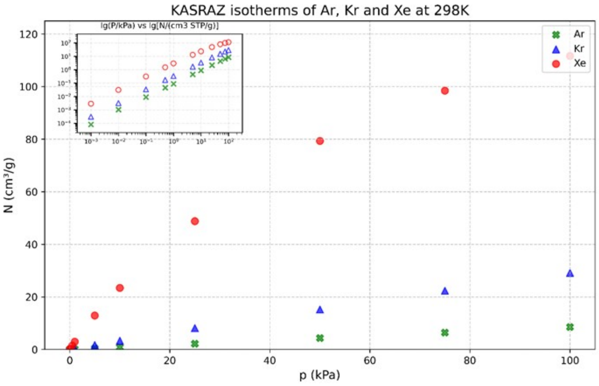

Figure S9. The GCMC simulated isotherms of adsorbates at 298K for KASRAZ (Cd).

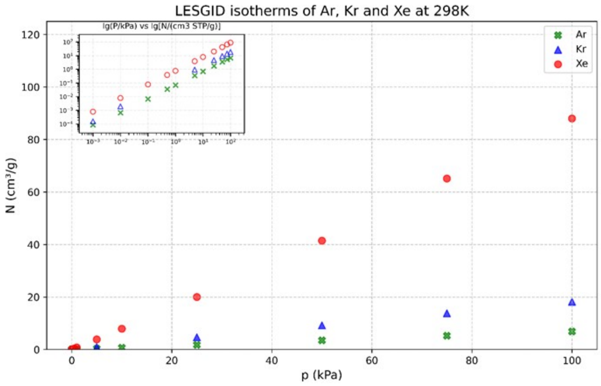

Figure S10. The GCMC simulated isotherms of adsorbates at 298K for LESGID (Zn).

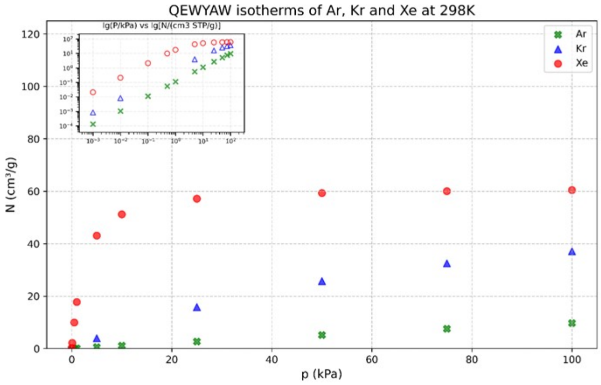

Figure S11. The GCMC simulated isotherms of adsorbates at 298K for QEWYAW (Cd).

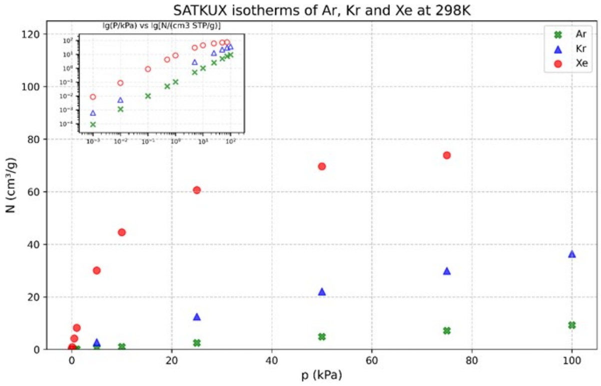

Figure S12. The GCMC simulated isotherms of adsorbates at 298K for SATKUX (Mn).

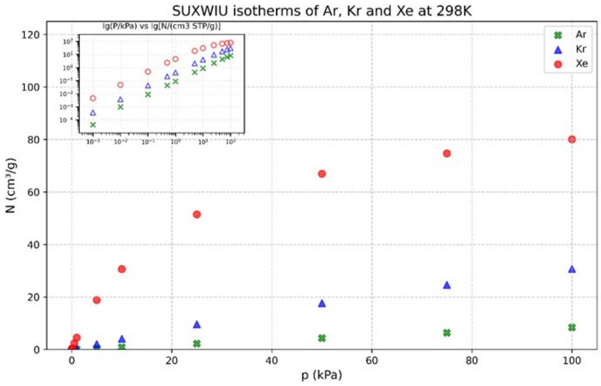

Figure S13. The GCMC simulated isotherms of adsorbates at 298K for SUXWIU (Ni).

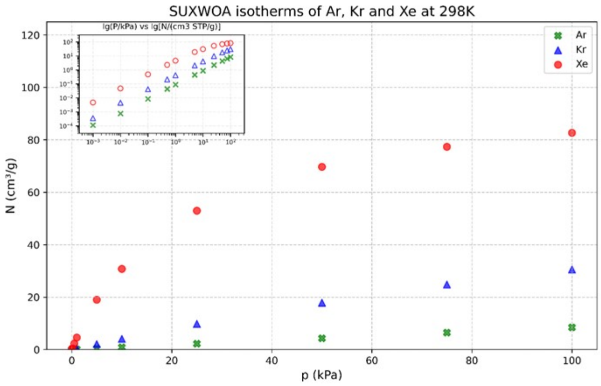

Figure S14. The GCMC simulated isotherms of adsorbates at 298K for SUXWOA (Co).

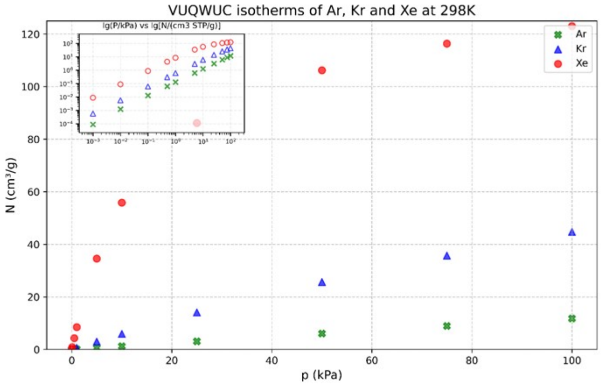

Figure S15. The GCMC simulated isotherms of adsorbates at 298K for VUQWUC (Mg).

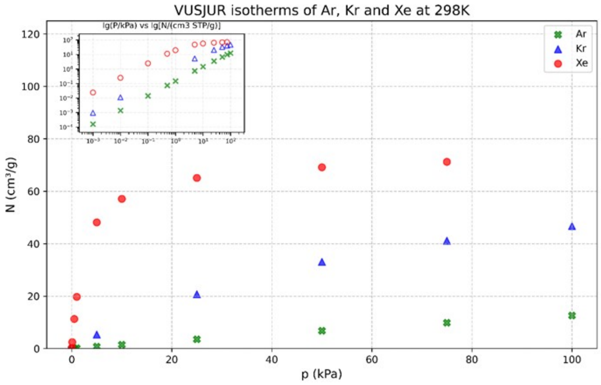

Figure S16. The GCMC simulated isotherms of adsorbates at 298K for VUSJUR (Zn).

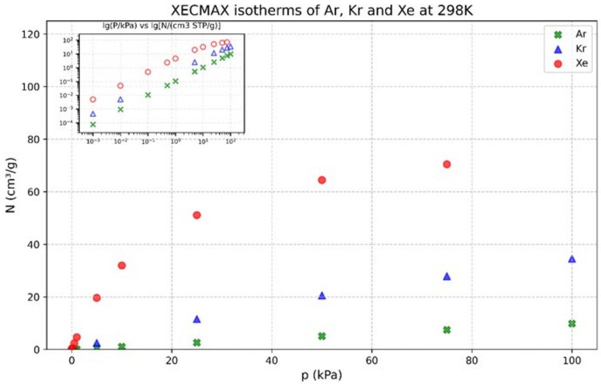

Figure S17. The GCMC simulated isotherms of adsorbates at 298K for XECMAX (Zn).

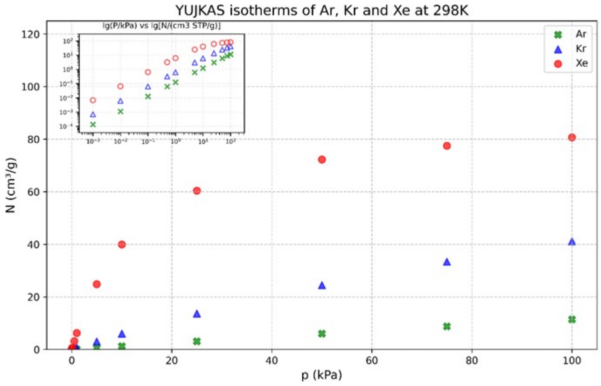

Figure S18. The GCMC simulated isotherms of adsorbates at 298K for YUJKAS (Mn).

S1.4 Topological details for structures of interest dataset.

Figure S19 illustrates the three-dimensional structures of the GF Set. The topological information was listed in Table S8.

Table S8. Topological information of the 18 frameworks in the GF Set. In point symbols, superscripts indicate the number of minimal rings each node participates in, subscripts denote node repetition within the network, braces {} enclose each node's ring combination, and dots "·" separate different ring sizes. This notation is obtained from the ToposPro software. CN means Connectivity Number. For the metal nodes, structures were listed the CNs of all metals at different chemical environment and noted as cluster to distinguish from single metal nodes.

RefCode (Metal)	Node Type	Ligand Node CN	Metal (Cluster) Node CN	Point Symbol
EKOBUE (Ca)	4,8-c	8	Ca: 4,4	$\{4^{10}\cdot6^{15}\cdot8^{3}\}\ \{4^{5}\cdot6\}_2$
ELEDEH (Nd)	6^2-c	6	Nd: 6	$\{4^{10}\cdot6^{5}\}\ \{4^{7}\cdot6^{8}\}$
HECXAS (Eu)	5,6,7,8-c	8	Eu: 5,6,7	$\{3\cdot4^{19}\cdot5^{2}\cdot6^{6}\}_2\ \{3^{2}\cdot4^{11}\cdot5^{6}\cdot6^{2}\}\ \{3^{2}\cdot4^{4}\cdot5^{2}\cdot6^{2}\}\ \{4^{11}\cdot6^{4}\}$
IZEQUB (Ni)	4,8-c	4	Ni cluster: 8	$\{4^{16}\cdot6^{12}\}\ \{4^{4}\cdot6^{2}\}_2$
JUJRIS (Sr)	4,8-c	8	Sr: 4	$\{4^{12}\cdot6^{12}\cdot8^{4}\}\ \{4^{6}\}_2$
KADYOH (La)	6^2-c	6	La: 6	$\{4^{10}\cdot6^{5}\}\ \{4^{7}\cdot6^{8}\}$
KAFHOS (Ce)	6^2-c	6	Ce: 6	$\{4^{10}\cdot6^{5}\}\ \{4^{7}\cdot6^{8}\}$
KASQUS (Zn)	4,8-c	8	Zn: 4,4	$\{4^{20}\cdot6^{8}\}\ \{4^{6}\}_2$
KASRAZ (Cd)	4^2,8-c	8	Cd: 4; Cd cluster: 4	$\{4^{12}\cdot6^{16}\}\ \{4^{4}\cdot6^{2}\}\ \{4^{5}\cdot6\}$
LESGID (Zn)	4,8-c	8	Zn: 4,4	$\{4^{20}\cdot6^{8}\}\ \{4^{6}\}_2$
QEWYAW (Cd)	3^2,6-c	6	Cd: 3,3	$\{4\cdot6^{2}\}\ \{4^{3}\}\ \{4^{4}\cdot6^{8}\cdot8^{3}\}$
SATKUX (Mn)	3,4,6,8-c	8	Mn: 6,6,4 (O: 3,3)	$\{4^{3}\cdot6^{2}\cdot8\}_2\ \{4^{3}\}_2\ \{4^{8}\cdot6^{10}\cdot8^{10}\}\ \{4^{8}\cdot6^{6}\cdot8\}$
SUXWIU (Ni)	4,8-c	8	Ni cluster: 4,4	$\{4^{20}\cdot6^{8}\}\ \{4^{6}\}_2$
SUXWOA (Co)	4,8-c	8	Co cluster: 4,4	$\{4^{20}\cdot6^{8}\}\ \{4^{6}\}_2$
VUQWUC (Mg)	4,8-c	8	Mg: 4,4	$\{4^{16}\cdot6^{12}\}\ \{4^{4}\cdot6^{2}\}_2$
VUSJUR (Zn)	5,6,8^2-c	6,8	Zn: 5; Zn cluster: 8	$\{3^{2}\cdot4^{6}\cdot5^{4}\cdot6^{3}\}\ \{3^{3}\cdot4^{11}\cdot5^{7}\cdot6^{7}\}_2\ \{3^{3}\cdot4^{3}\cdot5^{3}\cdot6\}_2\ \{3^{4}\cdot4^{10}\cdot5^{10}\cdot6^{3}\cdot7\}$
XECMAX (Zn)	3^2,4,5,6-c	5,6	Zn: 3,4,4,3	$\{4^{2}\cdot6\}\ \{4^{3}\cdot6^{3}\}\ \{4^{3}\}\ \{4^{5}\cdot6^{5}\}_2\ \{4^{8}\cdot6^{14}\cdot8^{6}\}$
YUJKAS (Mn)	4^2,8-c	8	Mn: 4,4	$\{4^{13}\cdot6^{14}\cdot8\}\ \{4^{5}\cdot6\}_2$

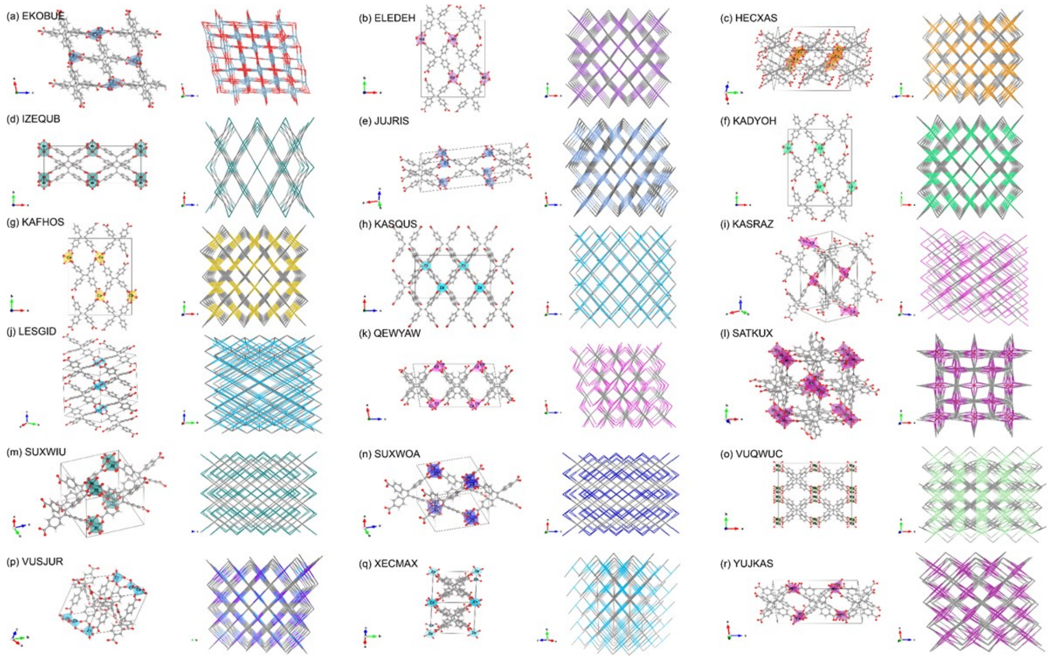

Figure S19. Crystal structural diagrams and simplified network diagrams of the 18 screened structures. (a)-(r) arranged in alphabetical order of their RefCodes (reference codes in Cambridge Structure Database).

Section S2. Laboratory Validation

S2.1 Materials

Cadmium nitrate tetrahydrate (AR, Sinopharm Chemical Reagent Co., Ltd.), zinc nitrate hexahydrate (AR, Sinopharm Chemical Reagent Co., Ltd.), cerium(IV) nitrate hexahydrate (AR, Sinopharm Chemical Reagent Co., Ltd.), lanthanum(III) nitrate hexahydrate (AR, Sinopharm Chemical Reagent Co., Ltd.), neodymium(III) nitrate hexahydrate (AR, Sinopharm Chemical Reagent Co., Ltd.), 1,1,2,2-tetra(4-carboxylphenyl)ethylene (98%, Jilin Chinese Academy of Sciences - Yanshen Technology Co., Ltd.), ethanol (≥99.7%, Sinopharm Chemical Reagent Co., Ltd.), N,N'-dimethylformamide (DMF) (≥99.0%, Sinopharm Chemical Reagent Co., Ltd.), acetone (≥99.5%, Sinopharm Chemical Reagent Co., Ltd.), and nitric acid (65~68%, Sinopharm Chemical Reagent Co., Ltd.) were purchased from the mentioned sources and used without further purification.

Helium, nitrogen, argon, krypton and xenon compressed gas tanks (reached the purity grade of 99.999 %) were provided from Shanghai Xiang Kun Special Gas Company for isotherm measurements.

S2.2 Preparation of Five Most Xenon-Selective Materials

Synthesis of QEWYAW. The synthetic procedure was scaled up directly in accordance with the original recipe.[1]

Synthesis of KAFHOS. Similarly, the synthetic procedure was scaled up directly in accordance with the original recipe.[2]

Synthesis of VUSJUR. The synthesis was simplified as follow.

2.8 mL $Zn(NO_3)_2 \cdot 6H_2O$ water solution (1 mol/L), 0.2 mL deionized water, 8 mL DMF, 0.3 mL HNO_3 acid (1 mol/L), and 4 mL H_4TCPE in DMF solution (0.02 mol/L) added in a 20-mL glass vial. The solution was sonicated to form well-mixed solution, sealed, and then heated in oven at 80 °C for 2 days.

Synthesis of KADYOH. Similar to the aforementioned, the synthetic procedure was scaled up in accordance with the original reference.[3]

Synthesis of ELEDEH. Similar to the aforementioned, the synthetic procedure was scaled up in accordance with the original reference.[4]

Solvent Replacement and Sample Activation. The as-synthesized samples were rinsed with distilled water (ELEDEH, Nd-MOF), or acetone (KADYOH, La-MOF), or ethanol (others), filtered on smooth nylon membrane, and then dried in vacuum oven at 80 °C overnight. The dried powders were stored for characterizations.

S2.3 Adsorption Test

Gas adsorption and desorption isotherms were performed (after vacuumed at 150 °C for at least 8 hours) by a BSD-660 series Advanced Specific Surface Area and Micropore Analyzer from BSD Instrument Technology (Beijing) Co., Ltd. Nitrogen isotherms were collected at 77 K in a liquid nitrogen bath dewar. Noble gas isotherms were tested at 298 K in an automated circulating water-bath system using an ethylene glycol–water mixture as the heat-transfer medium. To calculate the isosteric heats of adsorption (Q_{st}) for xenon, we tested at temperature of 273 K, 298 K and 313 K.

S2.3.1 Measured Isotherms

The noble gases isotherms for five most xenon-selective candidates were plotted as Figure S20-S24.

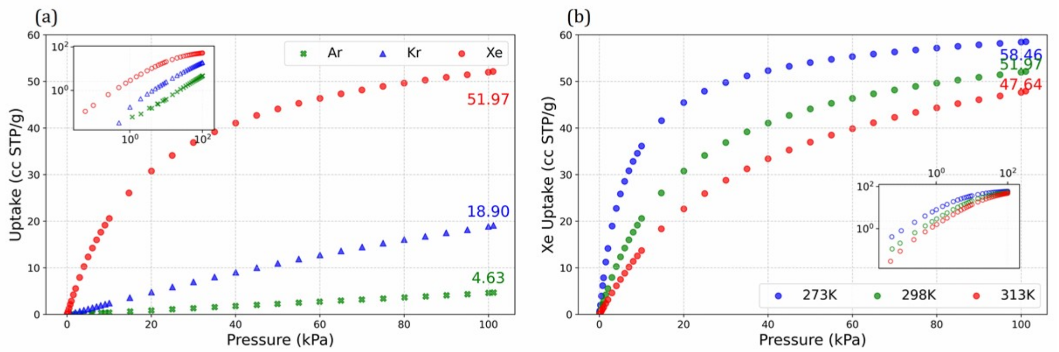

Figure S20. The measured isotherms of adsorbates for QEWYAW (Cd). (a) shows the isotherms of three single-component adsorbates and (b) shows the xenon isotherms at different temperatures.

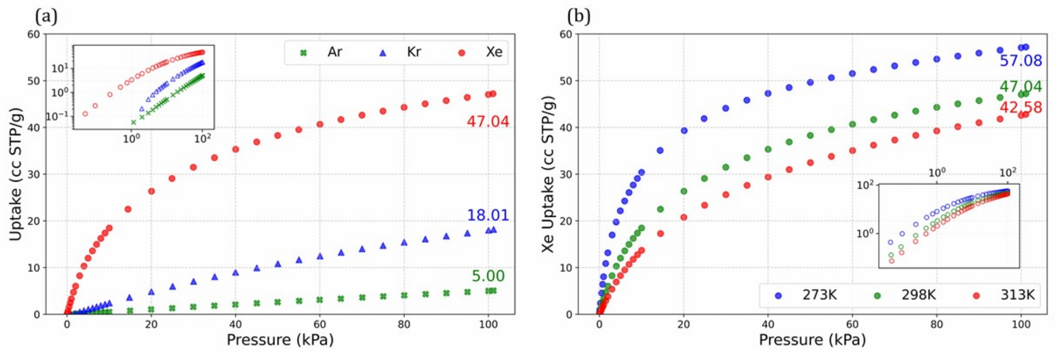

Figure S21. The measured isotherms of adsorbates for KAFHOS (Ce). (a) shows the isotherms of three single-component adsorbates and (b) shows the xenon isotherms at different temperatures.

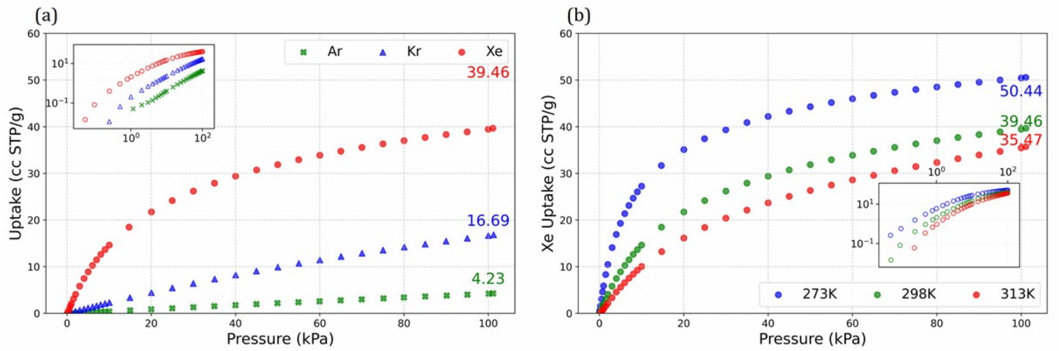

Figure S22. The measured isotherms of adsorbates for VUSJUR (Zn). (a) shows the isotherms of three single-component adsorbates and (b) shows the xenon isotherms at different temperatures.

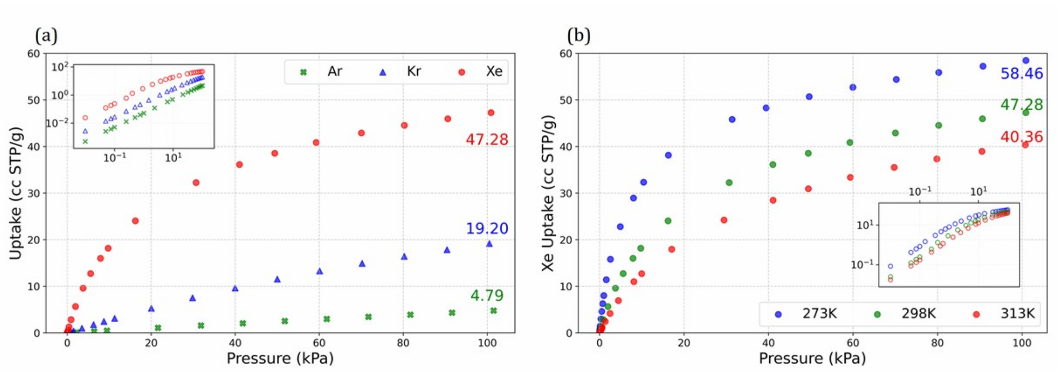

Figure S23. The measured isotherms of adsorbates for KADYOH (La). (a) shows the isotherms of three single-component adsorbates and (b) shows the xenon isotherms at different temperatures.

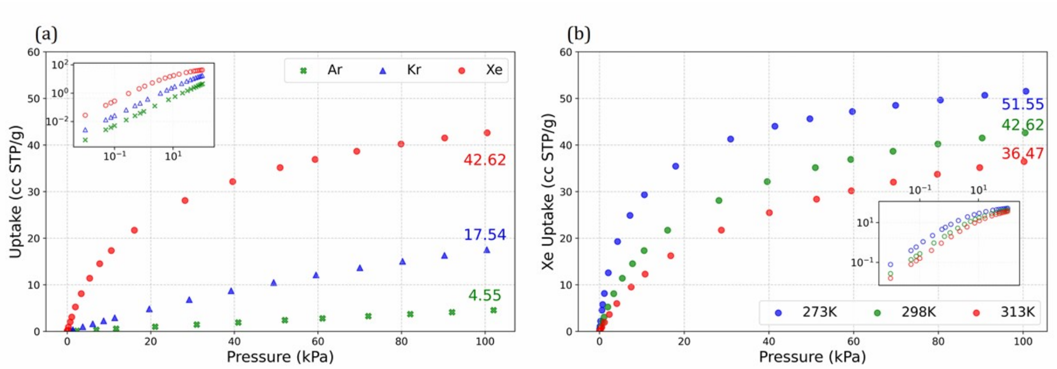

Figure S24. The measured isotherms of adsorbates for ELEDEH (Nd). (a) shows the isotherms of three single-component adsorbates and (b) shows the xenon isotherms at different temperatures.

Table S9. Best-fitting parameters for single-component isotherms at 298 K from measurement in lab. The model types and definitions of parameters were explained in Section 2.2 of the manuscript.

RefCode	Gas	Model type	GoF	A_1	A_2	B_1	B_2	C_1	C_2
QEWYAW (Cd)	Ar	SSLF	3.88	76.77	0	4.69E-04	1	1.06596	1
QEWYAW (Cd)	Kr	SSLF	4.66	58.28	0	3.85E-03	1	1.04766	1
QEWYAW (Cd)	Xe	DSLF	5.56	53.03	10.86	3.89E-02	7.77E-02	0.98513	1.2127
KAFHOS (Ce)	Ar	SSLF	4.85	37.21	0	1.27E-03	1	1.04331	1
KAFHOS (Ce)	Kr	SSLF	3.39	41.88	0	4.73E-03	1	1.09970	1
KAFHOS (Ce)	Xe	DSLF	5.93	52.44	13.62	2.49E-02	1.66E-01	0.93263	1.1017
KADYOH (La)	Ar	SSLF	5.59	90.27	0	5.86E-04	1	0.98712	1
KADYOH (La)	Kr	SSLF	5.89	57.05	0	5.22E-03	1	0.99222	1
KADYOH (La)	Xe	DSLF	5.09	51.02	12.24	2.76E-02	1.57E-01	0.95484	1.1263
ELEDEH (Nd)	Ar	SSLF	5.80	83.34	0	6.11E-04	1	0.98311	1
ELEDEH (Nd)	Kr	SSLF	5.78	51.40	0	5.47E-03	1	0.98696	1
ELEDEH (Nd)	Xe	DSLF	6.10	46.48	11.89	2.80E-02	1.55E-01	0.92980	1.1038
VUSJUR (Zn)	Ar	SSLF	4.05	26.56	0	1.33E-03	1	1.07624	1
VUSJUR (Zn)	Kr	SSLF	4.85	51.34	0	4.62E-03	1	1.00840	1
VUSJUR (Zn)	Xe	DSLF	4.83	45.90	13.81	2.55E-02	6.93E-02	0.86029	1.2359

Table S10. Comparison of uptakes and IAST selectivity from GCMC simulated and experimental measured results. All values are at 1 bar and 298 K if not specified. Uptakes are in cc/g STP units. Values marked with asterisk (*) for EKOBUE from experimental measurement were reported at 293 K instead. The experiment data of EKOBUE (Ca) and JUJRIS (Sr) are from their original references.

RefCode (Metal)	GCMC simulation					Experimental measurement						
	Uptake			$S_{Xe/Kr}$	$S_{Xe/Ar}$	Uptake					$S_{Xe/Kr}$	$S_{Xe/Ar}$
	Xe	Kr	Ar			Xe 273K	Xe 298K	Xe 313K	Kr	Ar		
QEWYAW (Cd)	60.4	37.1	9.71	32.2	203	58.5	52.0	47.6	18.90	4.63	11.36	60.80
ELEDEH (Nd)	53.9	40.8	11.8	26.6	267	51.6	42.6	36.5	17.54	4.55	9.74	61.35
KAFHOS (Ce)	54.2	41.0	12.1	26.2	254	57.1	47.0	42.6	18.01	5.00	11.47	62.15
KADYOH (La)	54.1	40.9	11.9	24.9	242	58.5	47.3	40.4	19.52	4.79	9.71	61.27
VUSJUR (Zn)	72.2	46.6	12.6	21.9	177	50.4	39.5	35.5	16.69	4.23	8.24	47.85
EKOBUE (Ca)	109	56.4	14.8	13.1	91.0	70.1	64.5*	60.1	34.3*	10.7*	7.4*	40.9*
JUJRIS (Sr)	87.9	41.6	10.5	10.8	69.2	-	~18.9	-	~6.8	-	~7	-

S2.3.2 Heat of Adsorption for Xenon

Figure S25 illustrated the heats of adsorption versus xenon uptake at 298 K for the five most xenon-selective MOFs.

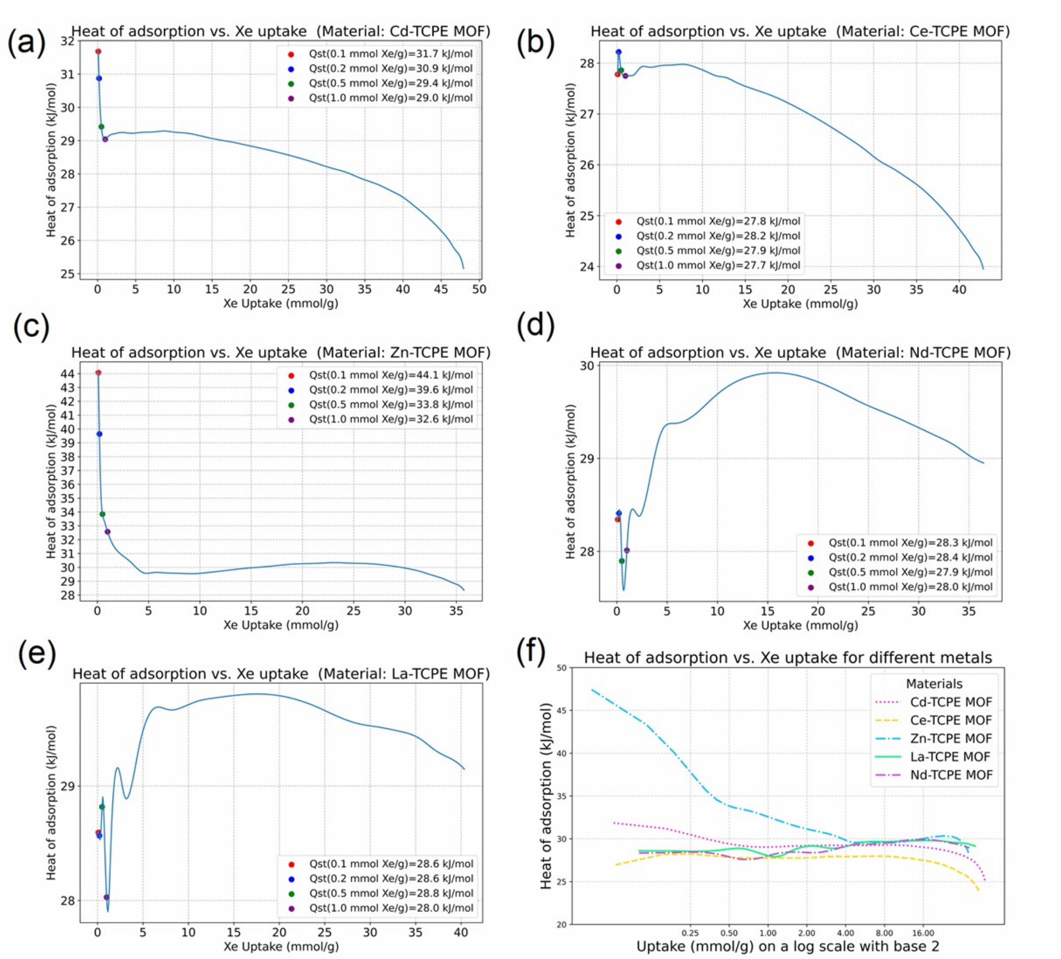

Figure S25. Heat of adsorption versus xenon uptake at 298 K for the five most xenon-selective MOFs. (a) QEWYAW (Cd), (b) KAFHOS (Ce), (c) VUSJUR (Zn), (d) ELEDEH (Nd), (e) KADYOH (La), and (f) all plotted on the same coordinate axes with uptake on a log scale with base 2.

S2.3.3 Pore Volume and Pore Size Distribution

Figure S26-S30 show the pore volume and pore size distribution by NLDFT for the five most xenon-selective MOFs.

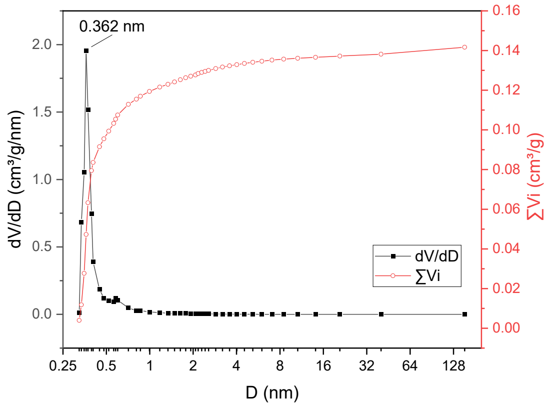

Figure S26. The logarithmic pore volume and pore size distribution by NLDFT for KAFHOS (Ce)

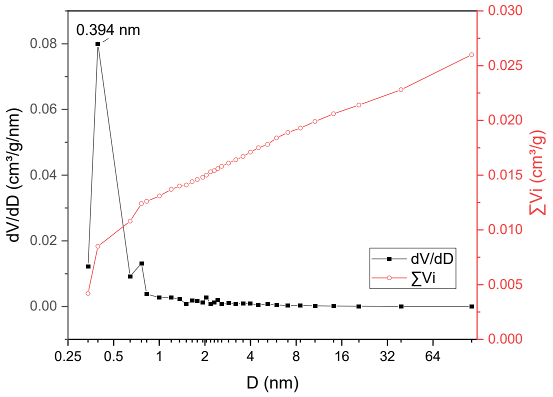

Figure S27. The logarithmic pore volume and pore size distribution by NLDFT for QEWYAW (Cd).

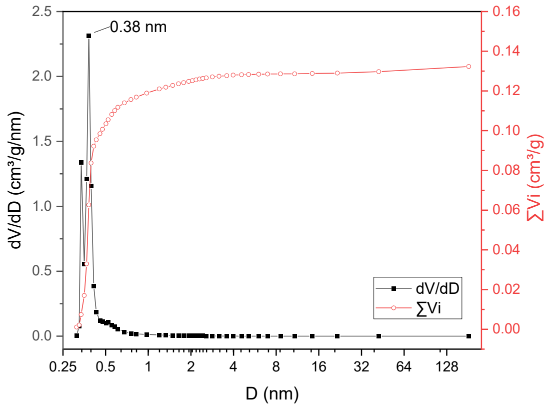

Figure S28. The logarithmic pore volume and pore size distribution by NLDFT for KADYOH (La).

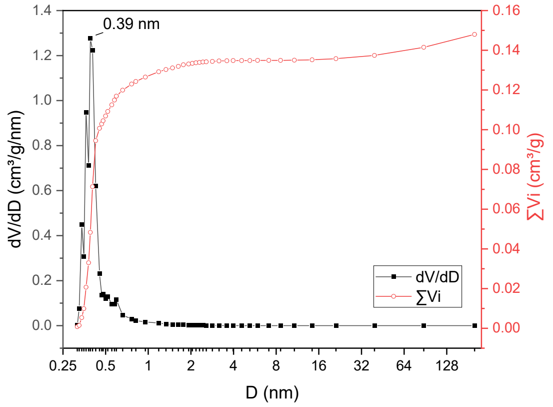

Figure S29. The logarithmic pore volume and pore size distribution by NLDFT for ELEDEH (Nd).

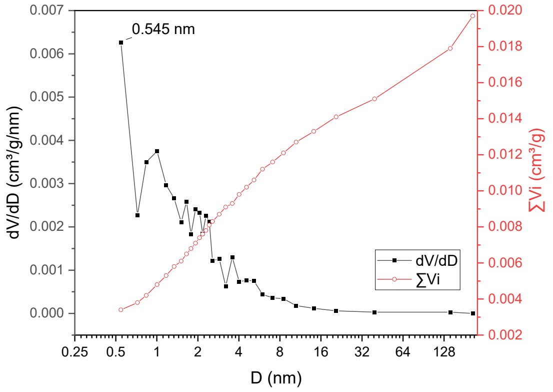

Figure S30. The logarithmic pore volume and pore size distribution by NLDFT for VUSJUR (Zn).

S2.4 Powder X-ray Diffraction Test

Powder X-ray diffraction (PXRD) measurements were recorded on a Bruker D8 ADVANCE diffractometer with Cu-Kα radiation (λ = 1.5418 Å) in the angular range 2θ scanned from 5° to 50° at ambient condition, operating at a voltage of 40 kV and a current of 40 mA. Figures S31-S35 showed the successful identification of as-synthesized powder materials with matching peak patterns from simulated patterns generated by Mercury software with CSD exported CIFs as inputs. Figure S36 showed PXRD patterns of KAFHOS demonstrating structural stability under ionizing radiation. The retention of peak positions and relative intensities across all irradiated samples confirms framework integrity under conditions relevant to nuclear off-gas processing.

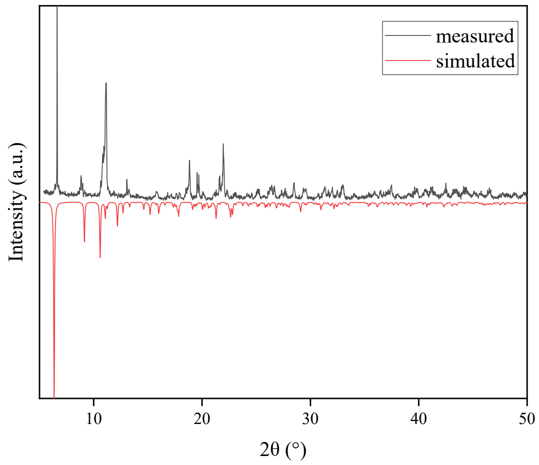

Figure S31. The powder X-ray diffractogram of as-synthesized (black line) and simulated QEWYAW (Cd) (red, reversed line).

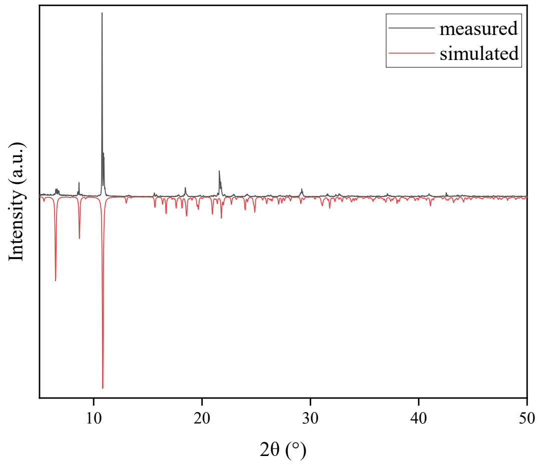

Figure S32. The powder X-ray diffractogram of as-synthesized (black line) and simulated KAFHOS (Ce) (red, reversed line).

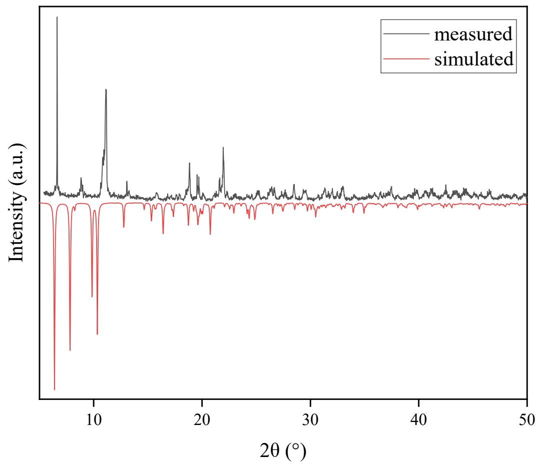

Figure S33. The powder X-ray diffractogram of as-synthesized (black line) and simulated VUSJUR (Zn) (red, reversed line).

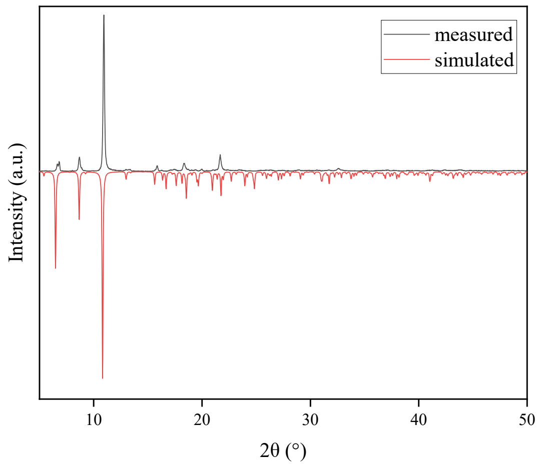

Figure S34. The powder X-ray diffractogram of as-synthesized (black line) and simulated ELEDEH (Nd) (red, reversed line).

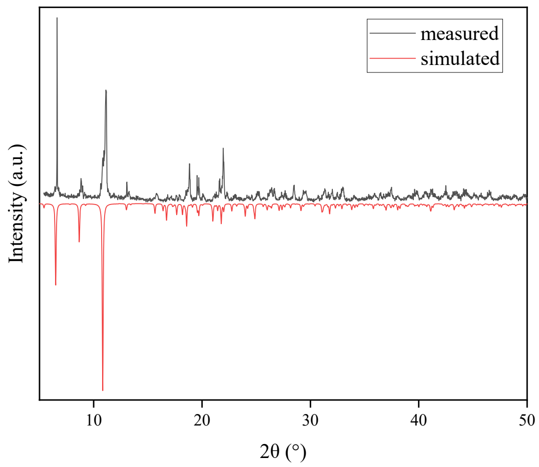

Figure S35. The powder X-ray diffractogram of as-synthesized (black line) and simulated KADYOH (La) (red, reversed line).

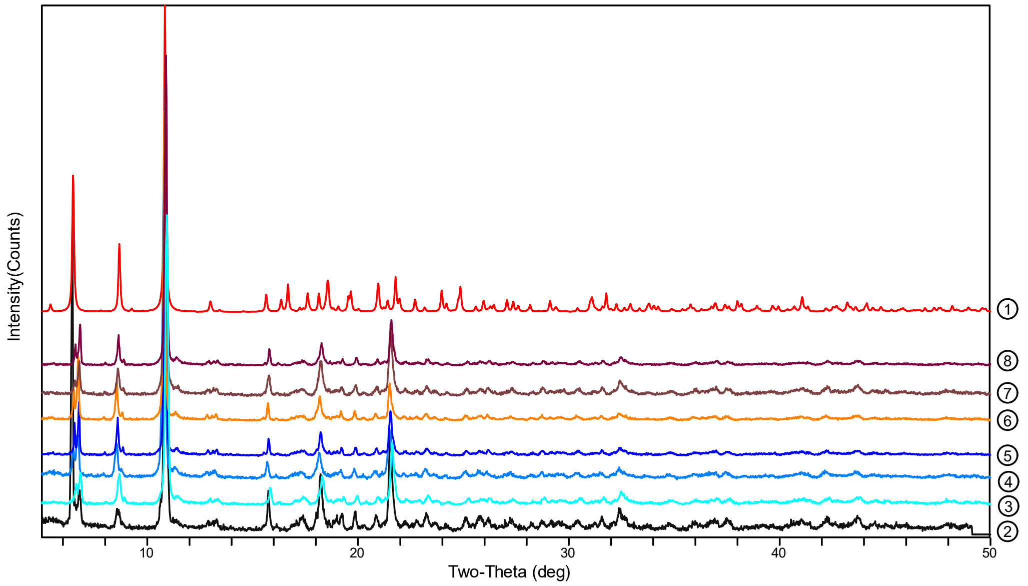

Figure S36. PXRD patterns of KAFHOS (Ce) demonstrating structural stability under ionizing radiation. ① Simulated pattern from KAFHOS CIF data; ② as-synthesized powder; ③–⑤ after β-irradiation at 50, 100, and 200 kGy, respectively; ⑥–⑧ after γ-irradiation at 50, 100, and 200 kGy, respectively.

S2.5 Evaluation of the Adsorbent Performance Indicator

Table S11. The calculation of Adsorbent Performance Indicator (API). EKOBUE (Ca) and JUJRIS (Sr) values were estimated from experiment of their original references at 293 K and 298 K, respectively.

RefCode (Metal)	API	$\alpha_{Xe/Kr}$	Xe uptake at 1 bar (cc STP/g)	Xe uptake at 0.1 bar (cc STP/g)	molecular weight of formula (g/mol)	Z	Volume of unit cell (Å³)	Particle density (g/cc)	Working Capacity (cc STP/cc)	Averaged Enthalpy of adsorption (kJ/mol)
**KAFHOS (Ce)**	24.45	11.47	47.04	3.23	646.6	4	3009.3	1.427	62.50	26.8
**QEWYAW (Cd)**	24.16	11.36	51.97	2.78	729.3	4	3607.7	1.342	66.03	28.3
**KADYOH (La)**	18.58	9.71	47.17	3.07	645.4	4	3001.4	1.428	62.96	29.5
**ELEDEH (Nd)**	16.88	9.74	42.57	2.86	650.7	4	3018.4	1.431	56.83	29.4
**VUSJUR (Zn)**	11.85	8.24	39.47	2.03	734.6	2	1853.0	1.316	49.27	30.1
**EKOBUE (Ca)**	10.07	7.4	62.8	27.8	636.6	4	3615.2	1.169	40.93	26
**JUJRIS (Sr)**	8.99	7.0	33.05	9.25	255.8	2	2109.1	1.322	31.46	21

S2.6 Comparison of KAFHOS performance with other representative materials

Table S12. Benchmark comparison of Xe/Kr separation performance for representative porous materials, including metal–organic frameworks, porous organic cages, and other new adsorbents. All selectivity values are reported as IAST values for a 20/80 (v/v) Xe/Kr mixture at 298 K and approximately 1 bar, unless otherwise specified.

Adsorbents	Xe uptake (mmol/g)	IAST Selectivity	*Isosteric heat of Xe adsorption* $Q_{st, Xe}$ (kJ/mol)	Reference
**KAFHOS***	2.1	11.47	26.8	This work
**QEWYAW***	2.32	11.36	28.3	This work
**KADYOH***	2.1	9.71	29.5	This work
**ELEDEH***	1.9	9.74	29.4	This work
**VUSJUR***	1.76	8.24	30.1	This work
HAT_Cl_a	1.91	15.5	33.2	Ref[5]
HOF-FJU-168a	3.49	22.0	32.5	Ref[6]
HIAM-103	1.39	8.3	24.5	Ref[7]
ETTA_Cl_a	1.43	9.7	34.6	Ref[8]
ETTA_Br_a	1.36	10.5	35.7	Ref[8]
ECUT-60	4.3	11.36	30	Ref[9]
MOF-Cu-H	3.19	16.7	33.4	Ref[10]
ATC-Cu	4.95	19.1	29.4	Ref[11]
BUT-422	3.19	22.5	33.2	Ref[12]
ZUL-530	3.13	20.5	30.5	Ref[13]
ZJU-74(a)-Pd	2.81	103.4	45.5	Ref[14]
CROFOUR-2-Ni	1.61	15.5	30.5	Ref[15]
CopzNi	3.3	14.0	38.5	Ref[16]
SBMOF-1	1.40	16	35	Ref[17]
ZUL-C1	3.34	11.7	25	Ref[18]
ZUL-C2	3.05	11.7	28	Ref[18]
$[Co_3(C_4O_4)_2(OH)_2]$	1.35	69.7	43.6	Ref[19]
FJU-55	1.41	10.0	39.4	Ref[20]
CC3	2.4	12.5	31.3	Ref[21]

S2.7 Gas Breakthrough Experiments

A multicomponent adsorption breakthrough curve analyzer (BeiShiDe Instrument Technology, Beijing, China) was employed for this measurement. The KAFHOS (Ce) powder sample was activated at 423 K for 12 h under dynamic vacuum. Subsequently, 1.1849 g of the activated sample was packed into a quartz column (81.9 mm packed length, 6 mm inner diameter) secured between two plugs of compacted quartz wool at both ends. The packed column was purged with helium at 423 K for several hours to ensure complete activation. A gas mixture (0.40 mol% Kr, 0.10 mol% Xe, balanced Ar to 100 mol%) was then introduced at a flow rate of 5 cc min^{-1} and total pressure of 1.01–1.07 bar. The column was allowed to cool to and equilibrate at 298 K within a thermostatic chamber. The effluent gas composition was continuously monitored by mass spectrometry until complete breakthrough was achieved (Figure S37). The distinct retention times for each gas demonstrate effective separation of the three components, with Xe retained longest, followed by Kr, and Ar eluting rapidly as the low-adsorbing carrier gas.

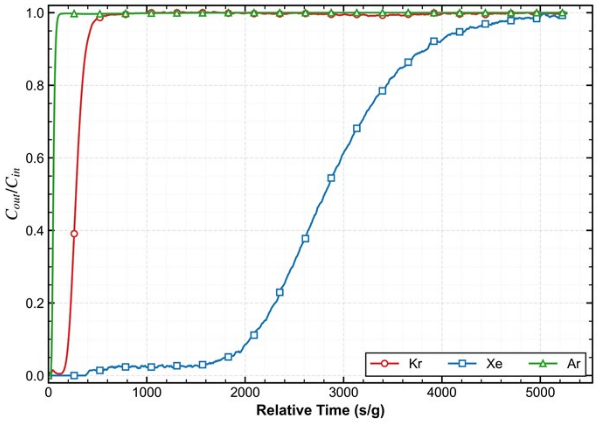

Figure S37. Dynamic breakthrough curves for Xe/Kr/Ar separation using KAFHOS (Ce) at 298 K and 1.01–1.07 bar. A ternary gas mixture (0.40 mol% Kr, 0.10 mol% Xe, balanced Ar) was flowed through the packed adsorbent bed at 5 cc min^{-1}. The effluent composition was monitored by mass spectrometry until complete breakthrough. The breakthrough points for Xe, Kr, and Ar are 1818.0 s/g, 187.0 s/g and 32.5 s/g, respectively.

References

1 H. Li, Y. Wang, F. Jiang, M. Li and Z. Xu, *Dalton Trans.*, 2023, **52**, 3846–3854.

2 Y. Dou, L. Yang, L. Qin, Y. Dong, Z. Zhou and D. Zhang, *J. Solid State Chem.*, 2021, **293**, 121820.

3 L. Yang, Y. Dou, L. Qin, L. Chen, M. Xu, C. Kong, D. Zhang, Z. Zhou and S. Wang, *Inorg. Chem.*, 2020, **59**, 16644–16653.

4 R. Huai, M. Xu, Y. Dou, Z. Wang, Z. Xue, Y. Zhang, H. Lv, L. Qin, D. Zhang, Z. Zhou and L. Yang, *Inorg. Chem. Commun.*, 2021, **127**, 108550.

5 Y. Xie, Q. Gao, J. Wang, G. Ye and B. Chen, *Angew. Chem. Int. Ed.*, 2025, **64**, e202509905.

6 L. He, Y. Li, L. Li, Z. Wang, Y. Chen, F. Yuan, G. Lan, C. Chen, S. Xiang, B. Chen and Z. Zhang, *Angew. Chem. Int. Ed.*, 2025, **64**, e202418917.

7 F.-A. Guo, K. Zhou, J. Liu, H. Wang and J. Li, *Precis. Chem.*, 2023, **1**, 524–529.

8 Y. Xie, X. Ding, J. Wang and G. Ye, *Angew. Chem. Int. Ed.*, 2023, **62**, e202313951.

9 H. Zhang, Y. Fan, R. Krishna, X. Feng, L. Wang and F. Luo, *Sci. Bull.*, 2021, **66**, 1073–1079.

10 S. Xiong, Y. Gong, S. Hu, X. Wu, W. Li, Y. He, B. Chen and X. Wang, *J. Mater. Chem. A*, 2018, **6**, 4752–4758.

11 Q. Liu, Y. Gong, B. Liu, S. Xiong, H.-M. Wen and X. Wang, *Chem. Eng. J.*, 2023, **453**, 139849.

12 Y.-L. Zhao, X.-Y. Li, X. Bai, M.-Z. Li and X. Zhang, *Chem. Eng. J.*, 2026, **527**, 171349.

13 X. Zhu, T. Ke, P. Han, Z. Zhang, Z. Bao, Y. Yang, Q. Ren and Q. Yang, *J. Am. Chem. Soc.*, 2024, **146**, 24956–24965.

14 J. Pei, X.-W. Gu, C.-C. Liang, B. Chen, B. Li and G. Qian, *J. Am. Chem. Soc.*, 2022, **144**, 3200–3209.

15 M. H. Mohamed, S. K. Elsaidi, T. Pham, K. A. Forrest, H. T. Schaef, A. Hogan, L. Wojtas, W. Xu, B. Space, M. J. Zaworotko and P. K. Thallapally, *Angew. Chem. Int. Ed.*, 2016, **55**, 8285–8289.

16 L. Guo, F. Zheng, Q. Xu, R. Chen, H. Sun, L. Chen, Z. Zhang, Q. Yang, Y. Yang, Q. Ren and Z. Bao, *Ind. Eng. Chem. Res.*, 2022, **61**, 7361–7369.

17 J. Qian, G. Chen, S. Xiao, H. Li, Y. Ouyang and Q. Wang, *RSC Adv.*, 2020, **10**, 17195–17204.

18 J. Zhou, T. Ke, F. Steinke, N. Stock, Z. Zhang, Z. Bao, X. He, Q. Ren and Q. Yang, *J. Am. Chem. Soc.*, 2022, **144**, 14322–14329.

19 L. Li, L. Guo, Z. Zhang, Q. Yang, Y. Yang, Z. Bao, Q. Ren and J. Li, *J. Am. Chem. Soc.*, 2019, **141**, 9358–9364.

20 L. Gong, Y. Liu, J. Ren, A. Al-Enizi, A. Nafady, Y. Ye, Z. Bao and S. Ma, *Nano Res.*, 2022, **15**, 7559–7564.

21 M. Miklitz, S. Jiang, R. Clowes, M. E. Briggs, A. I. Cooper and K. E. Jelfs, *J. Phys. Chem. C*, 2017, **121**, 15211–15222.
